# A bizarre theropod from the Early Cretaceous of Japan highlighting mosaic evolution among coelurosaurians

**DOI:** 10.1038/srep20478

**Published:** 2016-02-23

**Authors:** Yoichi Azuma, Xing Xu, Masateru Shibata, Soichiro Kawabe, Kazunori Miyata, Takuya Imai

**Affiliations:** 1 Institute of Dinosaur Research, Fukui Prefectural University, 4-1-1 Kenjojima, Matsuoka, Eiheiji, Fukui, 910-1195 Japan; 2 Fukui Prefectural Dinosaur Museum, 51-11 Terao, Muroko, Katsuyama, Fukui 911-8601, Japan; 3 Key Laboratory of Vertebrate Evolution and Human Origins of Chinese Academy of Sciences, Institute of Vertebrate Paleontology and Paleoanthropology, Chinese Academy of Sciences, Beijing 100044, China; 4 Gifu Prefectural Museum, 1989 Oyana, Seki, Gifu 501-3941, Japan

## Abstract

Our understanding of coelurosaurian evolution, particularly of bird origins, has been greatly improved, mainly due to numerous recently discovered fossils worldwide. Nearly all these discoveries are referable to the previously known coelurosaurian subgroups. Here, we report a new theropod, *Fukuivenator paradoxus*, gen. et sp. nov., based on a nearly complete specimen from the Lower Cretaceous Kitadani Formation of the Tetori Group, Fukui, Japan. While *Fukuivenator* possesses a large number of morphological features unknown in any other theropod, it has a combination of primitive and derived features seen in different theropod subgroups, notably dromaeosaurid dinosaurs. Computed-tomography data indicate that *Fukuivenator* possesses inner ears whose morphology is intermediate between those of birds and non-avian dinosaurs. Our phylogenetic analysis recovers *Fukuivenator* as a basally branching maniraptoran theropod, yet is unable to refer it to any known coelurosaurian subgroups. The discovery of *Fukuivenator* considerably increases the morphological disparity of coelurosaurian dinosaurs and highlights the high levels of homoplasy in coelurosaurian evolution.

There has recently been a great increase in our understanding of coelurosaurian evolution, bird origins in particular, through comparative studies about modern and fossil birds and non-avian dinosaurs^1^,^2^. In addition, numerous recently discovered fossils from multiple continents^3^,^4^,^5^,^6^,^7^ have aided our knowledge about evolution and the global distribution of non-avian coelurosaurian dinosaurs. Nevertheless, increased sampling from different regions of the world is desired to better understand the coelurosaurian dinosaurs’ detailed evolutionary history and tie it to their palaeobiogeography through time.

Terrestrial sedimentary units of the Mesozoic are not widely exposed in Japan, and, to date, dinosaur specimens have been limited in both absolute number and the quality of their preservation^8^. Nevertheless, the Kitadani Dinosaur Quarry of the Lower Cretaceous Tetori Group in Fukui, Japan (Fig. 1) represents one of the richest Cretaceous terrestrial localities in Japan, having produced numerous specimens of plants, bivalves, gastropods, fish, small mammals, turtles, crocodyliforms, avialan and non-avialan dinosaur eggshells^9^, various pterosaur, avialan, and non-avialan dinosaur ichnofossils^10^, and several dinosaur taxa including the allosauroid *Fukuiraptor kitadaniensis*^11^,^12^, titanosauriform *Fukuititan nipponensis*^13^, iguanodontian *Fukuisaurus tetoriensis*^14^, and hadrosauroid *Koshisaurus katsuyama*^15^.

The excavation in this quarry in the summer of 2007 resulted in the discovery of a specimen preserving the majority of a small-sized theropod skeleton, which represents the most complete non-avialan dinosaur specimen recovered in Japan. Whereas the specimen displays a few striking similarities to dromaeosaurids^16^, it in fact represents a bizarre, basally branching maniraptoran theropod with a large number of autapomorphies. Furthermore, it exhibits a combination of primitive and derived features seen amongst a variety of theropod groups, which bears substantially on the early evolution of coelurosaurian theropods. In the present paper, we describe the specimen and discuss its implications for understanding coelurosaurian evolution.

## Results

Systematic palaeontology

Dinosauria Owen, 1842

Theropoda Marsh, 1881

Maniraptora Gauthier, 1986

*Fukuivenator paradoxus* gen. et sp. nov.

### Etymology

“Fukui” refers to Fukui Prefecture in the central Japan, where the specimen was recovered; “venator”, a Latin word for hunter; the species name refers to the surprising combination of characters in this theropod dinosaur.

### Holotype

**Figure 1:**
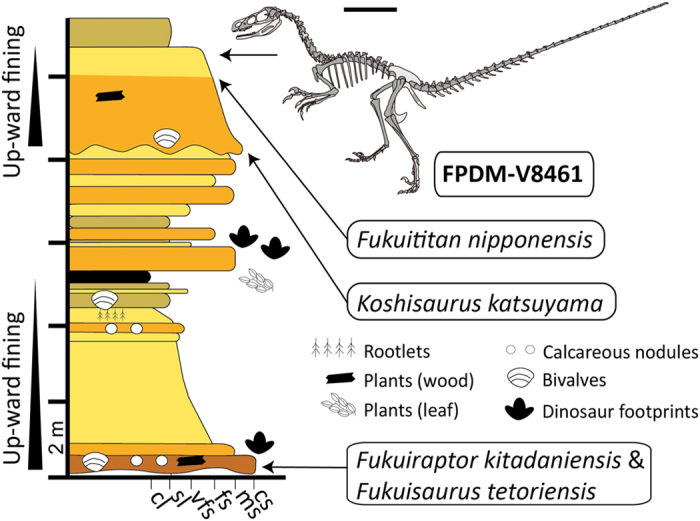
Skeletal silhouette of FPDM-V8461 and the stratigraphic section of the Lower Cretaceous Kitadani Formation in the Kitadani Dinosaur Quarry. Skeletal silhouette shows preserved bones in dark grey and missing bones in light grey. Positions of notable fossils including FPDM-V8461 are shown in the stratigraphic section. Scale bar = 50 mm. Abbreviations: cl, claystone; sl, siltstone; vfs, very fine sandstone; fs, fine sandstone; ms, medium sandstone; cs, coarse sandstone.

FPDM-V8461 (Fukui Prefectural Dinosaur Museum), a disarticulated but closely associated skeleton found within a 50 by 50 cm area, preserved elements include: incomplete right premaxilla with two isolated premaxillary teeth, left maxilla with one isolated and four intact teeth, left lacrimal, right jugal, right postorbital, left squamosal, both frontals, braincase, possible left ectopterygoid, left pterygoid, right palatine, posterior part of right dentary with two intact dentary teeth, eight cervical vertebrae, 10 dorsal vertebrae, five sacral vertebrae, and 30 caudal vertebrae, several cervical ribs, dorsal ribs, gastralia and chevrons, nearly complete scapulas and coracoids, most of both forelimbs, portions of both pubes, partial left ischium, and nearly complete hindlimbs (Fig. 1).

### Type locality and horizon

FPDM-V8461 was found in the Kitadani Dinosaur Quarry, which is on the Sugiyama River in the northern part of the city of Katsuyama, Fukui, Japan (36° 7′ 17.9″ N, 136° 32′ 41.4″ E) (see Supplementary Figs. S1 for quarry maps, and S2 for a field photograph). The quarry is locally referred to the Lower Cretaceous Kitadani Formation (Akaiwa Subgroup, Tetori Group). The age of the Kitadani Formation could be assigned to the Barremian to the Aptian on the basis of occurrences of the freshwater mollusk *Nippononaia ryosekiana*^17^ and charophytes^18^, and radioisotopic dates obtained from related sedimentary units (127–115 Ma?)^19^.

### Diagnosis

A relatively small theropod with the following unique features: 1. unusually large external naris (slightly smaller than antorbital fenestra in dorsoventral height); 2. large premaxillary fenestra subequal in size to maxillary fenestra; 3. large oval lacrimal pneumatic recess posterodorsal to the maxillary fenestra on antorbital fossa medial wall; 4. lacrimal with a distinct groove on lateral surface of anterior process and a ridge on lateral surface of descending process; 5. postorbital frontal process with T-shaped-cross section and laterally-flanged squamosal process; 6. an elongate tubercle on posterior surface of basal tuber of the basicranial region; 7. highly heterodont dentition featuring robust unserrated teeth including small spatulate anterior teeth, large and posteriorly curved middle teeth, and small and nearly symmetrical posterior teeth; 8. cervical vertebrae with a complex lamina system surrounding the neural canal resulting in deep and wide grooves for interspinous ligaments and additional deep sockets; 9. anterior cervical vertebrae with interprezygapophyseal, postzygadiapophyseal, prezygadiapohyseal, and interpostzygapophyseal laminae connecting to each other to form an extensive platform; 10. anterior and middle cervical vertebrae with transversely bifid neural spines; 11. dorsal, sacral, and anterior caudal vertebrae with strongly laterally curved hyposphene and centropostzygapophyseal laminae that, together with the postzygapophyseal facet, form a socket-like structure for receiving the prezygapophysis; 12. dorsoventrally bifurcated sacral ribs; 13. caudal zygapophyseal facets expanded to be substantially wider than the zygapophyseal processes; and 14. middle caudal vertebrae with transversely and distally bifid prezygapophyses.

### Description and comparison

FPDM-V8461 is probably a sub-adult individual based on some attributes of skeletal fusion in the skull and vertebrae (neurocentral synchondroses), though this is not a universal criterion for maturity across archosaurian taxa^20^. In particular, closed neurocentral sutures in nearly all vertebrae and sacral vertebrae completely fused to each other to form a sacrum are suggestive of adulthood, while the basisphenoid-parasphenoid complex unfused to the basioccipital and one posterior cervical neural arch remaining separate from the centrum suggest that FPDM-V8461 is not yet fully-grown. FPDM-V8461 is estimated to be 245 cm long including a reconstructed skull length of 23 cm (see Supplementary Information online), a preserved vertebral column length of 213 cm, plus an estimated missing vertebral column length of 9 cm. FPDM-V8461 is estimated to have a body mass of 25.0 kg when alive based on an empirical equation for estimating theropod body mass, similar to the predicated body mass for the ancestral Maniraptoriformes^21^.

**Figure 2:**
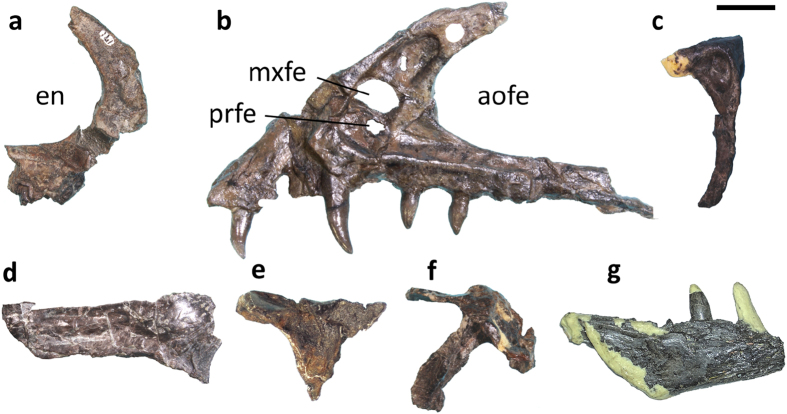
Cranial skeletal morphology of FPDM-V8461. (**a**) Partial right premaxilla in lateral view. (**b**) Partial left maxilla in lateral view. (**c**) Partial left lacrimal in lateral view. (**d**) Right frontal in dorsal view. (**e**) Right postorbital in lateral view. (**f**) Left squamosal in lateral view. (**g**) Partial right dentary in lateral view. Scale bar = 10 mm. Abbreviations: aofe, antorbital fenestra; en, external naris; mxfe; maxillary fenestra, prfe; premaxillary fenestra.

*Fukuivenator paradoxus* possesses a large number of unique features throughout the skeleton (see Diagnosis), but it also shares some striking derived features with other theropod groups. Many cranial features are shared with dromaeosaurids^16^,^22^,^23^. For example, the premaxilla has a broad subnarial shelf and a long maxillary process extending posteriorly to the level of about mid-length of the maxilla (a feature also present in ornithomimosaurs^24^), and the promaxillary fenestra is large (a feature also present in basally branching troodontids and *Archaeopteryx*^25^,^26^) (Fig. 2a). The maxillary fenestra is dorsally displaced (though not to the degree seen in dromaeosaurids^23^) and anteriorly located as in some dromaeosaurids such as *Linheraptor and Tsaagan*^23^,^27^ (Fig. 2b). The lacrimal is T-shaped in lateral view (a feature also present in troodontids and some other maniraptorans^16^,^22^,^23^,^27^,^28^,^29^) (Fig. 2c). The frontal bears a prominent notch along its anterolateral margin for receiving the lacrimal, and the supratemporal fossa has a sigmoid anterior border as in dromaeosaurids^16^,^30^ (Fig. 2d). Also similar to dromaeosaurids, the squamosal has an inset descending process below a lateral shelf^16^,^31^ (Fig. 2f). Unlike in dromaeosaurids but similar to troodontids, the dentary is sub-triangular in lateral view, and posterior teeth have proportionally short crowns^29^.

**Figure 3:**
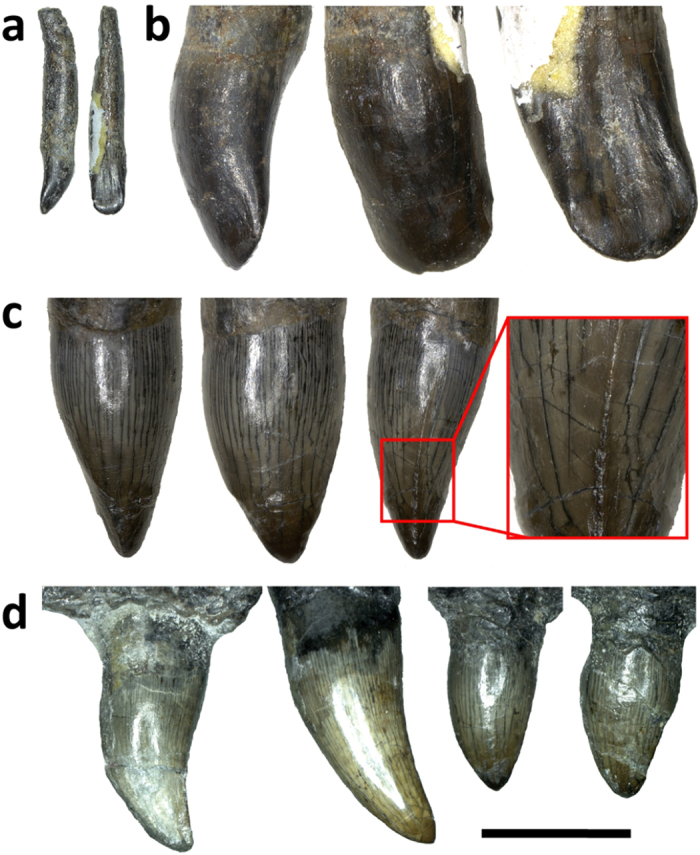
Premaxillary and maxillary teeth of FPDM-V8461. (**a**) Isolated anteriormost? premaxillary tooth in lateral (left) and posterior (right) views. (**b**) Enlarged view of the crown in (**a**) lateral (left), anterior (middle), and posterior (right) views. (**c**) Crown of an isolated left premaxillary tooth in distal (left), lateral (middle), mesial (right) views with an enlarged view of the mesial “ridge” (red box). (**d**) Intact left maxillary teeth in lateral view arranged as those in Fig. 2b. Scale bar = 10 mm for (**a,d**), 7.5 mm for (**b**), and 12.5 mm for (**c**).

Twelve alveoli are present and four maxillary teeth are intact with the maxilla. The teeth are unlike typical theropod teeth, particularly in terms of the absence of serrations and the presence of heterodonty^32^,^33^ (Fig. 3). All recovered teeth lack serrations on both mesial and distal carinae, and all except the anteriormost? premaxillary tooth have crowns with sub-oval cross sections, while they are variable in size and shape along the tooth row. The two premaxillary teeth differ substantially in size. The anteriormost? premaxillary tooth is small and somewhat spatulate (resembling to a degree the front teeth in the oviraptorosaur *Incisivosaurus*^34^,^35^). The other is leaf-like in lateral view and exhibits a “ridge,” on which denticles are absent, extending from the tip to the mid-section on the mesial surface. The anterior maxillary teeth have long crowns which are strongly curved posteriorly; in contrast, the posterior maxillary and posterior dentary teeth have leaf-like crowns with little posterior curvature. Unlike the premaxillary tooth, the “ridge” is absent in the maxillary teeth.

**Figure 4:**
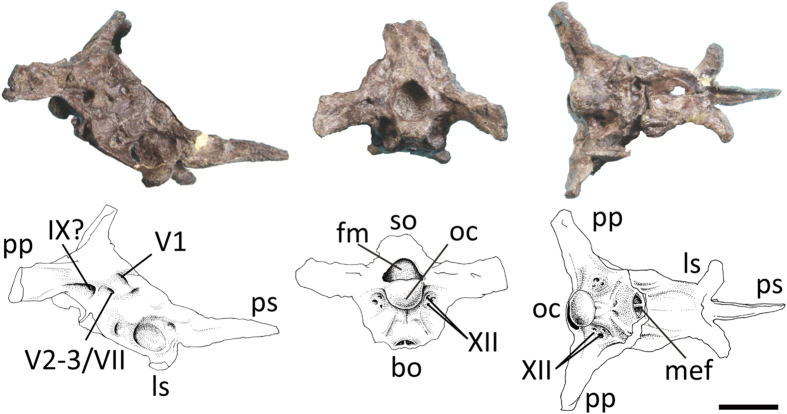
Braincase of FPDM-V8461 with interpretive drawings. Images in right lateral (left), posterior (middle) and ventral (right) views. Scale bar = 20 mm. Abbreviations: bo, basioccipital; fm, foramen magnum; ls, laterosphenoid; mef, medial eustachian foramen; oc, occipital condyle; pp, paroccipital process; ps, parasphenoid; so, supraoccipital. Roman numerals indicate positions of corresponding cranial nerves.

**Figure 5:**
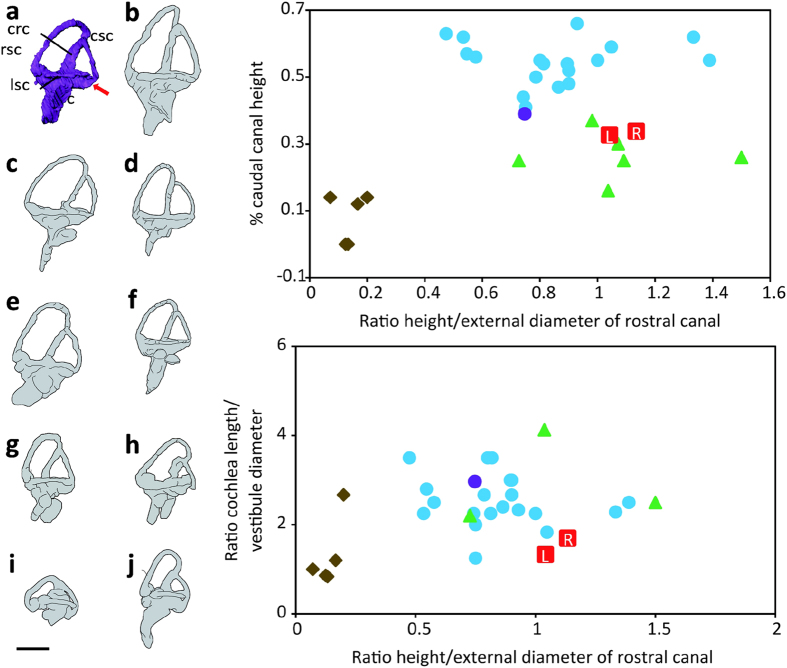
Comparisons of the inner ears of *Fukuivenator* and other selected animals. Inner ears of (**a**) *Fukuivenator paradoxus*, (**b**) *Allosaurus fragilis*, (**c**) *Tyrannosaurus rex*, (**d**) *Gorgosaurus libratus*, (**e**) *Struthiomimus altus*, (**f**) *Falcarius utahensis*, (**g**) *Deinonychus antirrhopus*, (**h**) *Archaeopteryx lithographica*, (**i**) *Gambelia wislizenii*, and (**j**) *Aythya fuligula*. Note that the caudal semicircular canal runs behind the lateral semicircular canal in (**a**) (red arrow). Plots: comparative proportions of the inner ears of Fukuivenator (red squares) against those of selected modern birds (blue circles), Archaeopteryx (purple circle), non-avian archosaurs (green triangles) and non-archosaur reptiles (brown diamonds), modified from Domínguez *et al*. 2004 (ref. 69). Scale bar = 20 mm for (**a**) and (**e**), 10 mm for (**b–d**) and (**f,g**), and 30 mm for (**h–j**). Abbreviations: % caudal canal height, height from the base of the caudal canal to the plane of the lateral canal/height of the caudal canal; c, cochlear duct; crc, crus communis; csc, caudal semicircular canal; lsc, lateral semicircular canal; rsc, rostral semicircular canal. References: (**a**) this paper; (**b–e,g,h**) Witmer & Ridgely, 2009 (ref. 37); (**f**) Lautenschlager *et al*., 2012 (ref. 70); (**i,j**) Walsh *et al*., 2009 (ref. 68).

In general, the braincase resembles closely that of relatively derived dromaeosaurids such as *Linheraptor* and *Velociraptor*^23^,^36^; the posterolaterally directed paroccipital process is long, its proximal half has a broad dorsal margin and its distal end is twisted anteriorly, the basipterygoid process is short and directs laterally and slightly ventrally, and the cultriform process extends anterodorsally (Fig. 4). As in troodontids^25^,^28^, the supraoccipitals form a broad, vertical eminence along the midline. We attempted to reconstruct a virtual brain endocast of *Fukuivenator* from computed-tomography (CT) images of the braincase of FPDM-V8461. The cerebrum region is poorly preserved due to flattening by a caudal vertebra on the anterior part of the braincase. Nevertheless, it was possible to digitally reconstruct the inner ears, including the semicircular canals and cochlea (Fig. 5a, also see Supplementary Information and Video online. Supplementary Table S1 online lists measurements of the inner ears). Each of the canals is planar and approximately circular, making the dorsal part of the labyrinth a more rectangular in lateral view, unlike the triangular shape in many non-avian theropods^37^,^38^,^39^. The rostral canal is the largest among the semicircular canals. It appears slender, bends backward and expands dorsally. As in birds, the caudal canal runs ventral to the surface of the horizontal canal and the cochlear duct extends ventrally. The cochlear duct also curves slightly medially, following the outline of the brain endocast. Semicircular canal proportions are rather more similar to those of non-avian archosaurs, while the cochlea morphologically resembles those of modern birds (Fig. 5).

**Figure 6:**
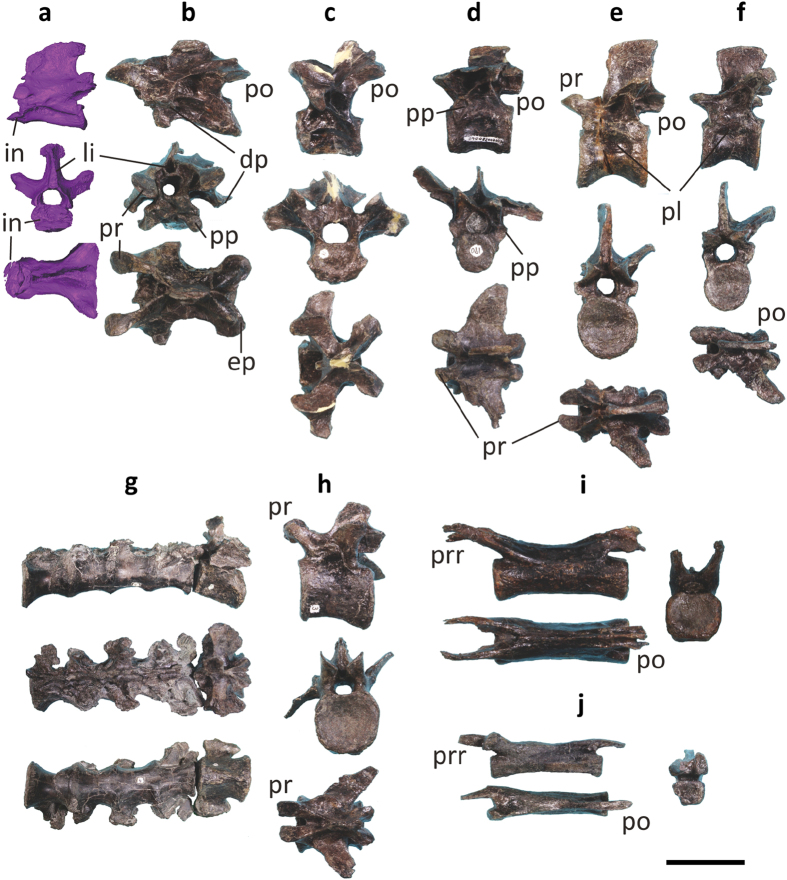
Postcranial axial skeletal morphology of FPDM-V8461. Anterior (**a**), middle (**b**), and posterior (**c**) cervical vertebrae in lateral (top), anterior (middle), and dorsal (bottom) views. Anterior (**d**), middle (**e**), and posterior (**f**) dorsal vertebrae in lateral (top), anterior (middle), and dorsal (bottom) views. (**g**) Sacral vertebrae in lateral (top), dorsal (middle), and ventral (bottom) views. Anterior (**h**), middle (**i**), and posterior (**j**) caudal vertebrae in lateral (top), anterior (middle), and dorsal (bottom) views. Scale bar = 25 mm for (**a–f,h**), 50 mm for (**g**), and 20 mm for (**i,j**). Note that the anterior cervical vertebra (**a**) is reconstructed based on CT-scanned images because it is severely fused to the braincase beyond preparation. Abbreviations: dp, diapophysis; ep, epipophysis; in, axis intercentrum; li, attachment scar of interspinal ligament; pl, pleurocoel; po, postzygapophysis; pp, parapophysis; pr, prezygapophysis; prr, prezygapophyseal rod.

Ten cervical vertebrae are preserved, missing at least the atlas, and thus *Fukuivenator* has at least 11 cervical vertebrae, which is greater in number than in most other non-avialan theropods^40^,^41^. The cervical centra are long anteroposteriorly and low dorsoventrally in lateral view, with the transverse width subequal to the dorsoventral height measured at the posterior articular surface (Fig. 6a–c). The centra of anterior cervical vertebrae are extremely angled so that their anterior articular surface is nearly in the same plane as the central ventral surface. The cervical vertebrae have highly developed and complex lamina system, particularly around the hyposphene-hypantrum region. In the anterior cervical vertebrae, the interprezygapophyseal, postzygadiapophyseal, prezygadiapohyseal, and interpostzygapophyseal laminae connect to each other to form an extensive dorsal platform (Fig. 6a). The middle cervical vertebrae have a highly modified hyposphene-hypantrum with the hypantrum extending ventrally below the dorsal border of the neural canal (Fig. 6b). The neural spines of cervical vertebrae are relatively long anteroposteriorly, though the posteriormost ones are short. The axial neural spine is transversely expanded distally to form a spine-table (Fig. 6a), and the neural spines of other anterior and middle cervical vertebrae are transversely bifid (Fig. 6b), a feature unknown in any other theropod yet present in some sauropods^42^. Axial ribs are present and they are much shorter than the axial centrum. Remaining cervical ribs are slightly longer than the vertebrae to which they articulate. The cervical ribs contain complex pneumatic fossae on the medial surface near the anterior end. Several cervical ribs have bifid anterior processes.

Ten dorsal vertebrae are preserved, including the anteriormost one. Dorsal centra are longer anteroposteriorly than tall dorsoventrally, unlike the dorsal centra of typical predatory theropods, which are much taller dorsoventrally than long anteroposteriorly (Fig. 6d–f). The central articular ends measure transversely subequal to dorsoventrally. No distinct hypapophysis is present, although the ventral surfaces of the posteriormost two cervical and anteriormost three dorsal vertebrae are sharply ridged, representing a weakly developed hypapophysis (Fig. 6d). Likely pleurocoels are present in all dorsal vertebrae in the form of longitudinal fossae on the lateral surfaces of centra, and they are more developed in the posteriormost dorsal vertebrae than in other dorsal vertebrae. Similar depressions are also seen in the anterior sacral vertebrae and even one anterior caudal centrum. The transverse processes of the dorsal vertebrae are relatively long and slender (more rod-like than strap-like), whereas the parapophyses of the dorsal vertebrae including the posterior ones are stalk-like as in derived alvarezsauroids and dromaeosaurids, though they are not as prominent as in the latter groups^23^,^40^,^43^. In most theropod groups, the parapophyses are flush with the dorsal central lateral surface. Similar to cervical vertebrae, the lamina system is well developed in the dorsal vertebrae. The hyposphene is strongly curved to form a semi-canal (i.e. open-half-cylinder in shape) with postzygapophyseal facet in the middle and posterior dorsal vertebrae (Fig. 6e–f). The neural spines of anterior and posterior dorsal vertebrae are expanded posteriorly at the distal end, but those of middle dorsal vertebrae are expanded both anteriorly and posteriorly.

Four fused sacral vertebrae and one isolated sacral are preserved. *Fukuivenator* is inferred to have at least six sacral vertebrae because the central articular surfaces of the isolated sacral do not match those of the remaining sacral vertebrae (the texture and surface topography of the articular surfaces on the isolated sacral also suggest that it would have fused to vertebrae both before and after it), suggesting that at least six sacral vertebrae were present. All sacral vertebrae are similar in size, unlike in most basally branching paravians where the middle ones are substantially larger than the other sacral vertebrae^44^,^45^ (Fig. 6g). The sacrum appears to be arched in lateral view. The ventral surface of the sacrum bears an extremely shallow longitudinal groove for most length (except the posterior half of the ventral surface of the last sacral centrum which is ridged). This longitudinal groove possibly represents the initial condition of the prominent ventral keel in the posterior sacral vertebrae of alvarezsauroids^46^. In most other theropod groups, the ventral surface of the sacral centra is rounded. The zygapophyses are fused to each other to form a platform lateral to the neural spines, a feature also known in dromaeosaurids^22^ but unreported in other theropod groups. The sacral-rib-transverse complex (fused sacral ribs and transverse processes) is strongly expanded dorsoventrally and bifurcated distally to contact the ilium, a feature previously unreported in any other theropod.

Thirty caudal vertebrae have been recovered, probably representing most of the caudal series, and thus the estimated total caudal number is approximately 35 as in some other basally branching coelurosaurian subgroups such as ornithomimosaurs^24^. The caudal vertebrae are variable in length through the sequence, and the middle ones are the longest (Fig. 6h–j), but proportionally not as long as those in basally branching paravians^45^. The articular ends of caudal centra are sub-rectangular in outline. In anterior caudal vertebrae, they are taller than wide, but in middle and posterior caudal vertebrae, they are wider than tall. A ventral sulcus is present in nearly every caudal centra, but they are variable in development along the series: relatively shallow in anteriormost and posterior caudal vertebrae and deep in anterior-middle and middle caudal vertebrae. The transverse processes are present in the anterior eight caudal vertebrae whereas absent in others. The transverse processes of the anteriormost caudal vertebrae are narrow at the distal end (Fig. 6h), different from those of many other groups such as dromaeosaurids in which the transverse processes are substantially expanded anteroposteriorly at the distal end^22^. Neural spines are present in nearly all preserved caudal vertebrae, although the posterior ones are extremely low. As in relatively basally branching tetanuran theropods, the neural spines of the anterior-middle caudal vertebrae are bifid longitudinally (Fig. 6i). The lamina system is still relatively well developed in caudal vertebrae and some anterior caudal vertebrae have postzygacentral laminae. The zygapophyses are highly modified; the zygapophyseal facets are located on the middle of the zygapophyseal process, substantially expanded transversely and larger than the neighbouring area, which is particularly true for the postzygapophyses. The most unusual feature is that the prezygapophyses of the middle caudal vertebrae are distally bifid (Fig. 6i), which has not been reported in any dinosaurs.

**Figure 7:**
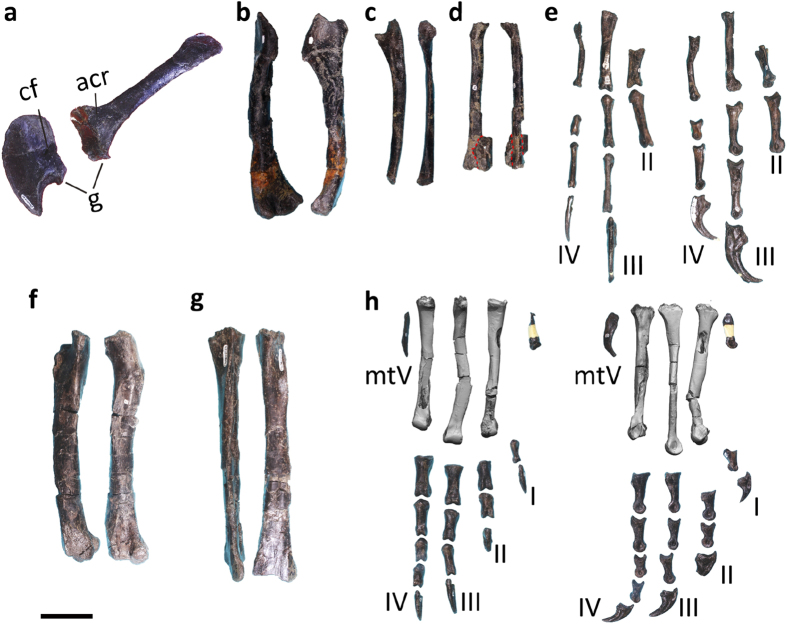
Appendicular skeletal morphology of FPDM-V8461. (**a**) Left coracoid (left) and scapula (right) in lateral view. (**b**) Left humerus in lateral (left) and posterior (right) view. (**c**) Left ulna in lateral (left) and posterior (right) view. (**d**) Left radius in lateral (left) and posterior (right) view (red dotted lines indicates the boundary between the radius and manual ungual? fused to each other). (**e**) Right manual elements in dorsal (left) and lateral (right) views. (**f**) Right femur in lateral (left) and posterior (right) view. (**g**) Left tibia in lateral (left) and anterior (right) views. (**h**) Right pedal elements in dorsal (left) and lateral (right) views. Scale bar = 50 mm. Note that the right metetarsals in (**h**) are reconstructed based on CT-scanned images because they are severely fused to each other. Abbreviations: acr, acromial process; cf, coracoid foramen; g, glenoid; mtV, metatarsal V.

The scapula and coracoid are in general similar to those of other basally branching maniraptorans. For example, as in therizinosaurs and many other maniraptoran groups^40^, the scapula and coracoid describe a sub-L-shape in lateral view (Fig. 7a). Also, as in some basally branching maniraptoriforms such as ornithomimosaurs^24^ and alvarezsaurs^46^, the scapula has a large acromion process of a squared-off profile and a small fossa located immediately anterodorsal to the glenoid fossa. On the other hand, the scapula is slightly shorter than the humerus, somewhat similar to those of paravian theropods where the scapula is considerably shorter than the humerus^47^. The coracoid is intermediate in outline between the basally branching maniraptoriforms and derived maniraptorans^45^; it has a long posteroventral process similar to those of ornithomimosaurs, whereas the ventral blade is wide and closer in condition to those of derived maniraptorans.

The forelimbs are about 75% of the hindlimb length, proportionally longer than those of most basally branching maniraptorans, but similar to those of therizinosauroids and more derived maniraptorans^48^. The humerus is relatively slender and curved anteriorly as in alvarezsauroids^49^ (Fig. 7b), in contrast to those of most theropod groups where the humerus is nearly straight in medial or lateral view. The medial margin near the proximal end of the humeral shaft is flat and broad and the deltopectoral crest expands slightly transversely near its distal end. There is a groove on the posterior surface of the deltopectoral crest close to the lateral edge, possibly for the insertion of the M. deltoideus. The distal end of the humerus is moderately expanded transversely, with the small radial condyle and larger ulnar condyle located on the anterior surface, and an anteriorly prominent ectepicondyle and a proximally located entepicondyle on the posterior surface. The ulna has a large olecranon process, a prominent coronoid process, and a posteriorly bowed shaft (Fig. 7c). The radius is slightly thinner than the ulna (Fig. 7d). Metacarpal II is about half the length of metacarpal III (We identify the three manual digits of maniraptoran theropods as II-III-IV following some studies^50^,^51^, although most theropod literature uses I-II-III numbering for the three manual digits of maniraptorans.) (Fig. 7e). The lateral condyle of metacarpal II only slightly exceeds the medial one in transverse width, such that the distal articular face is not as asymmetrical as that of most theropods, yet similar to that of derived ornithomimosaurs^24^. As in basally branching maniraptorans, metacarpal IV is considerably shorter than metacarpal III, and it is distinctly laterally bowed as in derived maniraptorans. As in ornithomimosaurs and alvarezsauroids, manual phalanx II-1 bears a pair of proximoventral heels separated by a longitudinal groove^24^,^49^. Manual phalanx IV-2 is short and has prominent proximoventral heel as in basally branching dromaeosaurids and troodontids^22^,^25^. The three manual unguals are considerably variable in morphology. Manual ungual II has a deep proximal end with a prominent proximodorsal lip, a small proximally located flexor tubercle, and tip extending strongly ventrally. Manual ungual III is only slightly longer than manual ungual II, but has a much shallower proximal end, a more distally located flexor tubercle, and a less ventrally extended tip. Manual ungual IV is much smaller than other unguals, and it has a prominent and distally located flexor tubercle and a highly arched profile with minimal ventral extension of the tip.

The pelvis is represented by a partial pubes and a partial left ischium. The sub-cylindrical pubic shaft bears a long pubic apron extending from its anterior edge. The ischium is similar to those of non-pennaraptoran theropods in having a rod-like ischial shaft^51^.

Similar to many other skeletal elements, the femur also exhibits a combination of primitive and derived features. As in basally branching maniraptorans^52^,^53^, the femur has a relatively deep cleft separating an alariform lesser trochanter from an anteroposteriorly narrow greater trochanter (Fig. 7f). In nearly all derived maniraptorans except *Utahraptor*^54^,^55^, the lesser trochanter has a cylindrical cross section and is separated from the greater trochanter by a small groove. However, the femur of *Fukuivenator* has a mound-like lateral ridge and its fourth trochanter is represented by a very low sharp ridge, similar to the conditions in those of derived maniraptorans^56^,^57^. The distal end of the femur is somewhat similar to basally branching alvarezsauroids in having a distally extended lateral condyle^49^. Metatarsal I is attached to the medial surface of metatarsal II, which has a semi-circular proximal articular end (Fig. 7h). The distal end of metatarsal III is only slightly ginglymoid. Metatarsal IV, as in derived maniraptorans, has a triangular proximal articular end. Metatarsal V is short and considerably curved. Pedal digit I is short and relatively robust as in therizinosauroids^58^; pedal digit II is more robust than other pedal digits, and it is similar to those of some basally branching paravians including deinonychosaurs in having a prominent dorsal extension of the distal end of phalanx II-1 and an enlarged ungual^25^; pedal digit III is only slightly longer than pedal digits II and IV, which are close to each other in length. Proximal pedal phalanges are similar to those of basally branching coelurosaurs such as the tyrannosauroid *Guanlong* in having paired proximoventral heels^59^. All pedal unguals are moderately curved.

**Figure 8:**
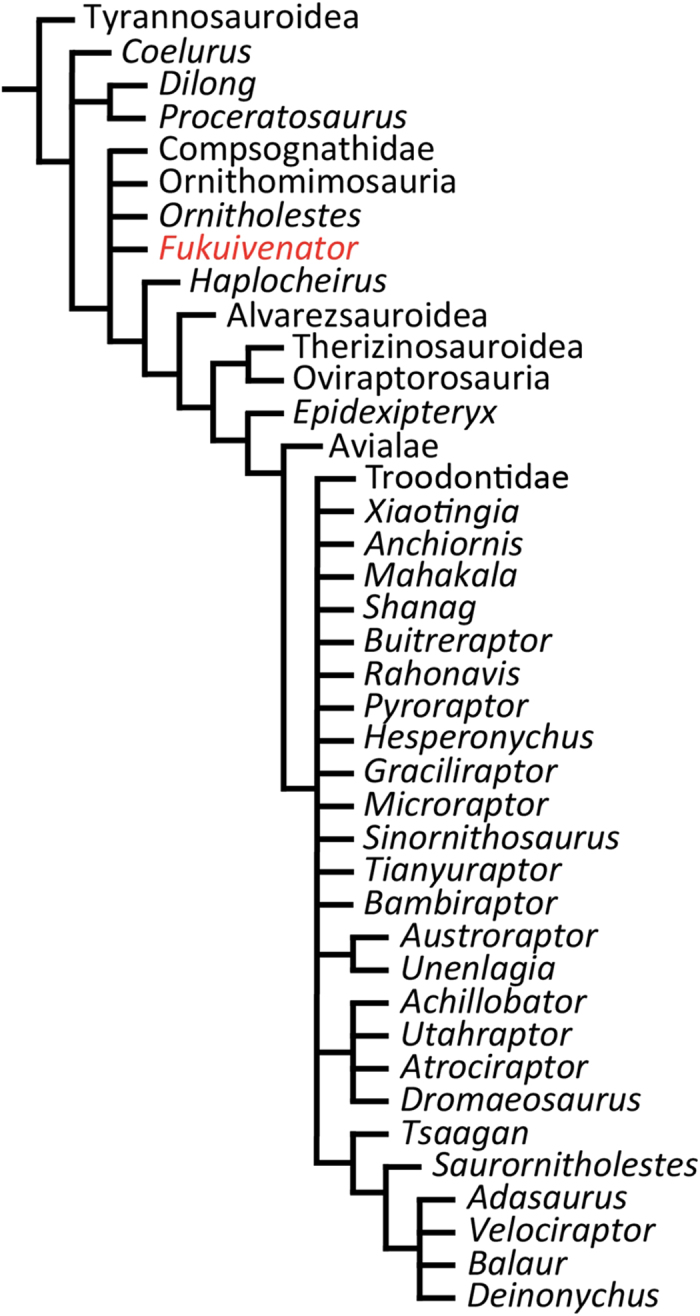
Suggested systematic position of *Fukuivenator paradoxus* within the Coelurosauria.

In order to assess the systematic position of *Fukuivenator*, we added this taxon to a recently published dataset for the coelurosaurian phylogeny^16^. Notable results derived from the analysis of the expanded matrix include: 1. *Fukuivenator* recovered as a basally branching maniraptoran, 2. the Therizinosauridae placed as the sister taxon to the Oviraptorosauria, 3. *Haplocheirus* placed outside the Alvarezsauroidea and *Anchiornis* and *Xiaotingia* outside the Troodontidae, and 4. the collapse of several coelurosaurian clades including the Dromaeosauridae (Fig. 8; see Supplementary Information and Supplementary Figs. S3 and S4 online for details). Admittedly, our phylogenetic hypothesis is only weakly supported by the available data, as indicated by some statistical metrics (see Supplementary Information and Supplementary Fig. S5 online).

Character optimization indicates that *Fukuivenator* lacks the synapomorphies of a clade comprising *Haplocheirus*, the Alvarezsauroidea, the Therizinosauroidea, the Oviraptorosauria, and the Paraves: e.g., maxillary and dentary teeth small and lesser trochanter separated from greater trochanter by a small groove. Furthermore, the analysis indicates that *Fukuivenator* lacks the synapomorphies of a clade comprising the Alvarezsauroidea, the Therizinosauroidea, the Oviraptorosauria, and the Paraves: e.g., basipterygoid process ventrally or anteroventrally projecting, prefrontal absent, pubic posteriorly oriented, and lesser trochanter of femur cylindrical in cross section. Character optimization also indicates that *Fukuivenator* lacks the synapomorphies of a clade comprising the Therizinosauroidea, the Oviraptorosauria, and the Paraves: e.g., olecranon process weakly developed and ischium with plate-like shaft.

Although *Fukuivenator* is recovered as a basally branching maniraptoran in our analysis, *Fukuivenator* shares many derived features with the Paraves, notably the Dromaeosauridae. To evaluate alternative systematic positions for *Fukuivenator*, we re-ran the analyses with several different constraints. Placing *Fukuivenator* in various alternative systematic positions within the Paraves requires from 3 additional steps up to 13 additional steps (see Supplementary Information and Supplementary Table S1 online), and in particular, placing *Fukuivenator* within the Dromaeosauridae requires only 3 additional steps. Character optimization also indicates that these derived features are independently evolved in *Fukuivenator* and other theropod groups, the Dromaeosauridae in particular. The discovery of a basally branching maniraptoran such as *Fukuivenator* with a large number of derived features seen in the crownward Deinonychosauria highlights the mosaic evolution and prevalence of homoplasies among the Coelurosauria.

## Discussion

Recent discoveries worldwide have added greatly to the diversity of the Coelurosauria^1^,^2^,^3^,^4^,^5^,^6^,^7^. Although these discoveries have substantially improved our understanding of the evolution of the clade, particularly of the origins of birds^1^,^2^, nearly all recent discoveries are referable to the known coelurosaurian subgroups^1^,^2^,^3^,^4^,^5^,^6^,^7^, with the exception of the bizarre Scansoriopterygidae^60^, a group possibly belonging to basally branching Oviraptorosauria^1^,^56^. Consequently, these discoveries have not substantially altered the overall phylogenetic framework for reconstructing the morphological, functional, and ecological evolution of the Coelurosauria. In contrast, *Fukuivenator* represents a bizarre coelurosaurian not assignable to any known coelurosaurian subgroup.

Among known coelurosaurian theropods, *Fukuivenator* appears similar to dromaeosaurids with respect to its numerous derived features. These include a maxillary fenestra located anteriorly and displaced dorsally, a frontal with an anterolateral notch for the lacrimal articulation, a sigmoidal anterior border to the supratemporal fossa, a lateral shelf on the squamosal overhanging the descending process, a distally anteriorly twisted paroccipital process, and a specialized second pedal digit. Some of these features are previously only known in dromaeosaurids and some in both dromaeosaurids and several other derived maniraptoran groups. However, *Fukuivenator* is clearly more basally branching than dromaeosaurids given its numerous plesiomorphic features: non-rectangular coracoid, ulna with prominent coronoid process, metacarpal IV considerably shorter than metacarpal III, femur with an alariform lesser trochanter and an anteroposteriorly narrow greater trochanter, and ischium with a rod-like shaft.

In addition to its unusual combination of derived and plesiomorphic features, *Fukuivenator* also possesses a large number of morphological features unknown in any other theropod, such as unusually large external naris, heterodont dentition with unserrated teeth, transversely bifid neural spines in cervical series, bifurcated sacral ribs, and distally bifid prezygapophyses in caudal vertebrae. Taken together, these unusual features of *Fukuivenator* expand the morphological disparity of theropod dinosaurs.

The inner ear morphology of *Fukuivenator* also exhibits a mosaic condition. The semicircular canal proportions suggest that *Fukuivenator* probably possessed the spatial sensory perception equivalent to that of cursorial non-avian theropod dinosaurs. On the other hand, the auditory ability of *Fukuivenator* might be comparable to that of modern birds, on the basis of a suggested correlation between the length of cochlea duct and hearing ability in modern birds^61^.

Dietary diversification may represent a major driver for morphological and functional diversity in theropod evolution^62^,^63^. A major dietary change appears to have occurred in the early evolution of maniraptoriforms^23^, indicated by a proportionally small head, long neck, and leaf-like, unserrated dentition in basally branching ornithomimosaurs. Our new discovery provides further evidence for such a scenario. Typical theropod teeth have posteriorly curved, blade-shaped, and serrated crowns, whereas those of many coelurosaurs have proportionally shorter and more symmetrical crowns without serrations. The dentition of *Fukuivenator* seems to represent an intermediate condition.

The vertebral morphology of *Fukuivenator* is also suggestive of a body-plan shifting away from a typical theropod, with a longer neck and vertebrae with a more complex lamina system. Proportionally long neck appeared multiple times independently through theropod evolutionary history, as seen in the possible ceratorsaur *Elaphosaurus*^64^, the tetanuran *Chilesaurus*^65^ and the Spinosauridae^66^ outside the Maniraptoriformes. It is notable that, in many cases, the elongation of neck appears associated with acquisition of unique teeth in different theropod groups (e.g., the Spinosauridae^66^, the Ornithomimosaurs^24^, and some members of the Troodontidae^29^), possibly reflecting dietary shift from typical carnivory.

No direct evidence (e.g., gut contents) for the diet of *Fukuivenator* is present. However, such characters of *Fukuivenator* as conical, unserrated premaxillary teeth, symmetrical maxillary teeth, heterodonty, and the number of cervical vertebrae >10 are consistent with the evidence of theropod herbivory^63^, suggesting at least omnivorous diet of *Fukuivenator*.

Our phylogenetic analysis recovers *Fukuivenator* as a basally branching maniraptoran, consistent with the general suite of morphological features it displays. The mosaic character distribution in *Fukuivenator* substantially impacted the coelurosaurian phylogeny. The large number of homoplasies introduced by *Fukuivenator* to the phylogenetic dataset collapsed several coelurosaurian clades including the Therizinosauroidea and Paraves.

The systematic position of *Fukuivenator* and its aforementioned morphological features suggest that this new taxon is substantial in coelurosaurian evolution. It documents a critical stage in the shift from a carnivorous diet to a more omnivorous one, and a transition which may contribute in part to the high diversity of coelurosaurian theropods.

## Methods

### Phylogenetic analysis

We added *Fukuivenator paradoxus* to the data set published by Turner *et al*. (2012) (ref. 16; see Supplementary Information). The matrix was analysed using TNT v. 1.0^67^ with the character ordering and weighting the same as in ref. 16. The analysis was conducted by default setting except for the following additional parameters: maximum number for trees in memory = equal to 100000, 1000 replicates, and 10 trees per replicate. We used the traditional search. The analysis produced 400 most parsimonious trees of 2195 steps (RI = 0.71; CI = 0.28), found in 40 out of the 1000 replications of RAS + TBR. Additional TBR branch swapping of these 400 trees found more than 100000 additional optimal topologies, resulting in a total of more than 100000 most parsimonious trees.

### CT scanning

The braincase, one anterior cervical vertebra and right metatarsals were scanned using an industrial microfocus CT, TXS320-ACTIS (TESCO Co., Yokohama, Japan), at FPDM. Voxel sizes for the three elements (the braincase, cervical vertebra, and metatarsals) ranged from 0.08721 mm (x and y axes) to 0.05 mm (z axis), from 0.0560156 mm (x and y axes) to 0.05 mm (z axis) and from 0.0698242 mm (x and y axes) to 0.10 mm (z axis), respectively. Acquisition parameters were 260–270 kV, 223–250 μA, a pixel size of each image of 1024 × 1024, and a slice thickness of 0.1–0.2 mm.

## Additional Information

**How to cite this article**: Azuma, Y. *et al*. A bizarre theropod from the Early Cretaceous of Japan highlighting mosaic evolution among coelurosaurians. *Sci. Rep.* **6**, 20478; doi: 10.1038/srep20478 (2016).

## Supplementary Material

Supplementary Information

Supplementary Information
