# Supplementary material for: A bizarre theropod from the Early Cretaceous of Japan highlighting mosaic evolution among coelurosaurians
Source: Sci Rep. 2016 Feb 23;6:20478. doi: 10.1038/srep20478 (PMC4763874; doi:10.1038/srep20478)
Supplement: Supplementary Information [file srep20478-s1.pdf]

Supplementary information to

A bizarre theropod from the Early Cretaceous of Japan highlighting mosaic evolution among coelurosaurians

Yoichi Azuma[1,2], Xing Xu[3], Masateru Shibata[1,2], Soichiro Kawabe[4], Kazunori Miyata[1,2], Takuya Imai[2]

[1]Institute of Dinosaur Research, Fukui Prefectural University, 4-1-1 Kenjojima, Matsuoka, Eiheiji, Fukui 910-1195, Japan
[2]Fukui Prefectural Dinosaur Museum, 51-11 Terao, Muroko, Katsuyama, Fukui 911-8601, Japan
[3]Key Laboratory of Vertebrate Evolution and Human Origins of Chinese Academy of Sciences, Institute of Vertebrate Paleontology and Paleoanthropology, Chinese Academy of Science, Beijing 100044, China
[4]Gifu Prefectural Museum, 1989 Oyana, Seki, Gifu 501-3941, Japan

1. **Quarry map and field photographs of the Kitadani Dinosaur Quarry**
2. **Selected measurements of FPDM-V8461**
3. **Supplementary information for the virtual brain endocast of FPDM-V8461**
4. **Phylogenetic analysis**
5. **Reference**

1. Maps and a field photograph of the Kitadani Dinosaur Quarry

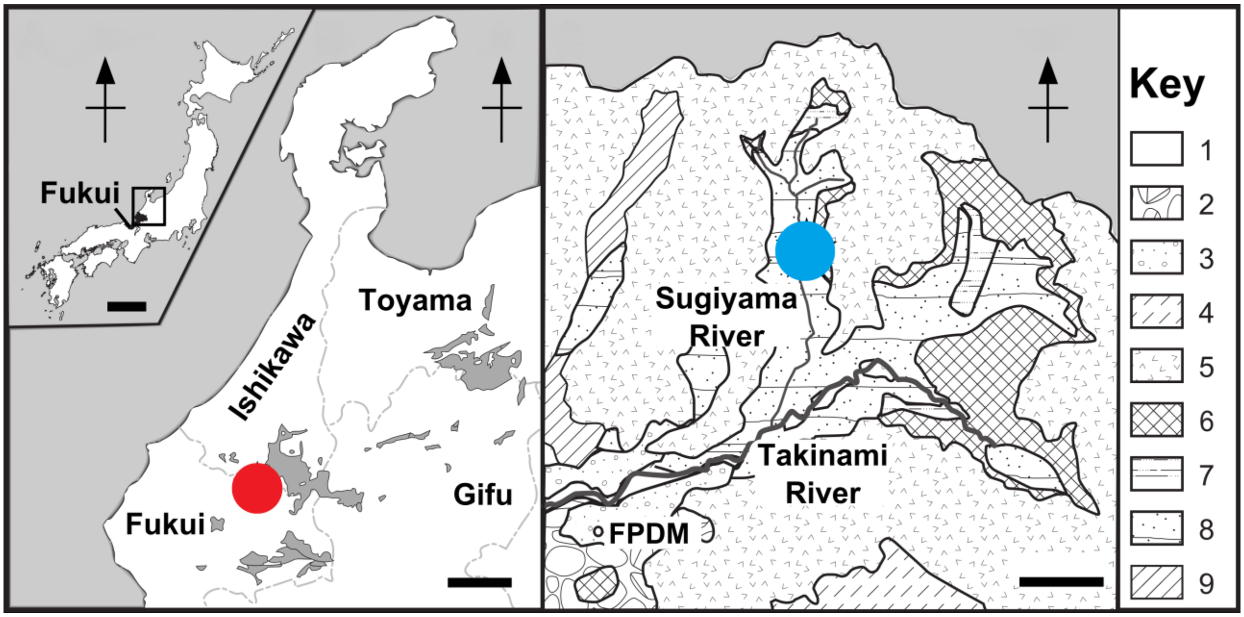

Figure S1 | Maps of the Kitadani Dinosaur Quarry. Upper left: location map of Fukui, Japan, scale bar = 200 km. Left: location map of Katsuyama, Fukui, Japan, indicated by a red circle, scale bar = 20 km. Right: geological map of the Kitadani Dinosaur Quarry, Katsuyama, indicated by a blue circle, Scale bar = 4 km. Keys: 1, alluvial deposits; 2, fan delta deposits; 3, terrace deposits; 4, glassy andesitic rocks; 5, andesitic rocks; 6, Nohi rhyolite; 7, Omichidani Formation; 8, Tetori Group; 9, Hida Metamorphic Rocks. FPDM = Fukui Prefectural Dinosaur Museum. Modified from Shibata & Goto (2008).

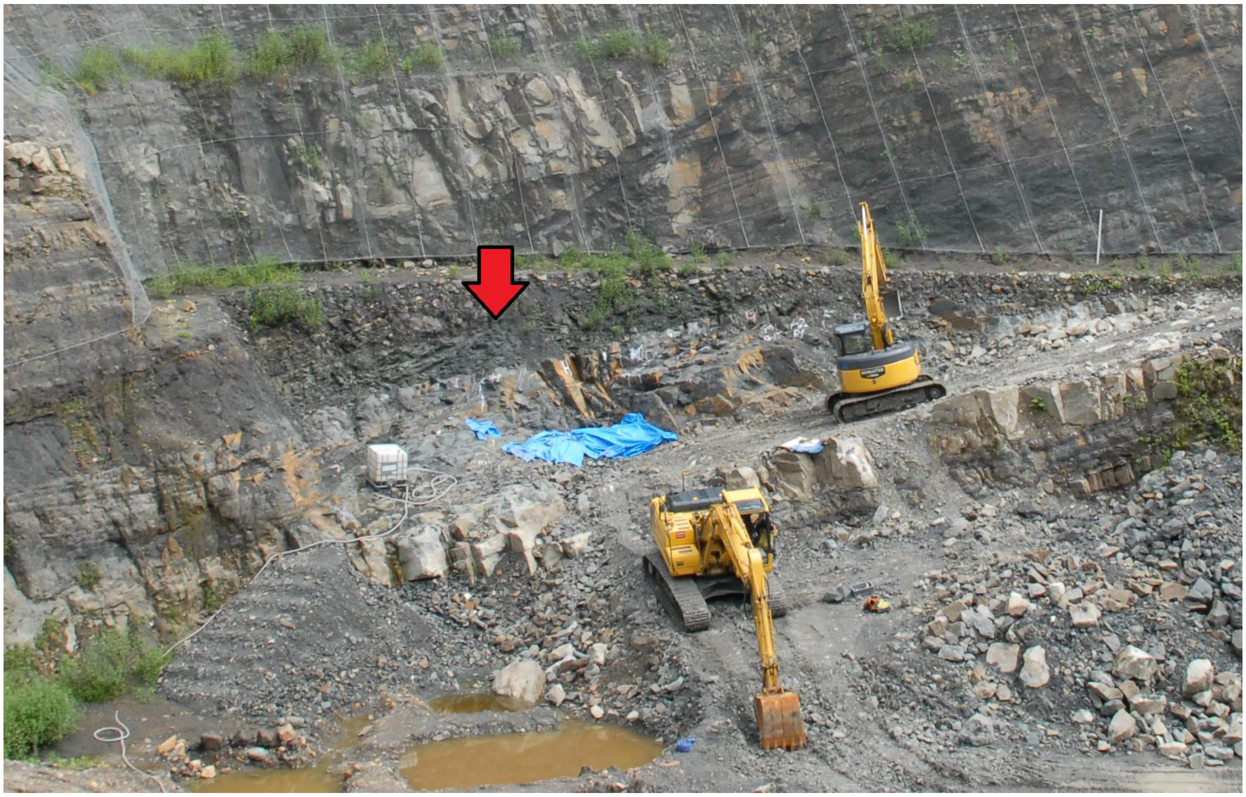

Figure S2 | Field photograph of the Kitadani Dinosaur Quarry. Red arrow indicates the horizon from which FPDM-V8461 was collected.

2. Selected measurements of FPDM-V8461

Measurements are in mm. Those with * are estimated values.

Skull length	234*
Pre-orbital length	126*
Antorbital fossa length	58*
Cervical series length	33*
Dorsal series length	35*
Sacral series length	13*
Caudal series length	133*
Scapula length (left/right)	132/128
Humerus length	134*
Ulna length	123*
Radius length	106*
Mc II length (left/right)	32/31
Mc III length (left/right)	63/?
Mc IV length (left/right)	51/?
Manual phalanx II-1 length (left/right)	47/?
Manual phalanx II-2 length (left/right)	55*
Manual phalanx III-1 length (left/right)	?/42
Manual phalanx III-2 length (left/right)	47/47
Manual phalanx III-3 length (left/right)	69*
Manual phalanx IV-2 length (left/right)	17/?
Manual phalanx IV-3 length (left/right)	36/?
Ischium length	164*
Femur length (left/right)	?/187
Tibia length	227*
Metatarsal I length (left/right)	?/30
Metatarsal II length (left/right)	?/110
Metatarsal III length (left/right)	?/116
Metatarsal IV length (left/right)	?/103
Metatarsal V length (left/right)	?/32
Pedal phalanx I-1 length (left/right)	20/18
Pedal phalanx II-1 length (left/right)	?/24
Pedal phalanx II-2 length (left/right)	21/?
Pedal phalanx III-1 length(left/right)	32/32

Pedal phalanx III-2 length (left/right)	?/29
Pedal phalanx III-3 length (left/right)	25/25
Pedal phalanx IV-1 length (left/right)	34/33
Pedal phalanx IV-2 length (left/right)	?/18
Pedal phalanx IV-3 length (left/right)	19/19
Pedal phalanx IV-4 length (left/right)	18/18

3. Supplementary information for the virtual brain endocast of FPDM-V8461

Explanation for Supplementary Video | CT slice images of the braincase of FPDM-V8461. The images are shown from the posterior end to the anterior end of the braincase. Note that a caudal vertebra adhering to the braincase appears on the top left in the video.

Table S1 | Measurements (mm) and proportions of the right and left inner ears of FPDM-V8461. Measurements were taken by the rendering software AMIRA (Mercury Computer Systems, San Diego, CA, USA) based on CT image stacks. Explanations: H. Rostral Canals, rostral canal height; D. Rostral Canals, rostral canal external diameter; % Caudal Canal Height, height from the base of the caudal canal to the plane of the lateral canal/height of the caudal canal; L. Cochlea, cochlea length measured from the tip to front of oval window; D. Cochlea Tubes, cochlea tube diameter at the front of oval window.

	H. Rostral Canals	D. Rostral Canals	% Caudal Canal Height	L. Cochlea	D. Cochlea Tubes
Right	9.67	8.52	0.33	7.45	4.40
Left	9.50	9.12	0.32	5.87	4.43

4. Phylogenetic analysis

The data matrix was analyzed using the TNT software package (Goloboff et al. 2008) . The analyses were run using a traditional search strategy, with default settings apart from the following: 300000 maximum trees in memory and 1000 replications. The analysis produced 100000 most parsimonious trees (tree length = 2080, RI = 0.71; CI = 0.28), the strict consensus of which is shown in Supplementary Fig. S3.

This strict consensus tree differs from the one produced in the analysis excluding *Fukuivenator* from the dataset (Supplementary Fig. S4) in several respects: *Haplocheirus* is placed outside the Alvarezsauroidea and *Xiaotingia* and *Anchiornis* outside the Troodontidae; the Therizinosauroidea is placed as the sister taxon to the Oviraptorosauria; the monophyly of the Dromaeosauridae is collapsed

We also ran Bremer support and bootstrap analyses on the data matrix, and Bremer support and bootstrap values for the recovered clades are indicated in Supplementary Fig. S5. It is notable that very few clades are strongly supported.

Given that *Fukuivenator* also displays a large number of derived features seen in derived maniraptorans, and in particular, it is extremely similar to dromaeosaurids, we re-run the analyses with several different constraints, including forcing *F. paradoxus* as a member of the Paraves, the sister taxon to the Paraves, a member of the Deinonychosauria, the sister taxon to the Deinonychosauria, a member of the Dromaeosauridae and the sister taxon to the Dromaeosauridae (Supplementary Table S2). Placing *Fukuivenator* in various alternative systematic positions within the Paraves requires from 3 additional steps up to 13 additional steps. In particular, placing *Fukuivenator* within the Paraves, the Deinonychosauria, or the Dromaeosauridae requires only three additional steps, suggesting that the placement of *Fukuivenator* at the base of the Maniraptoria is not strongly supported by our dataset.

We listed all the synapomorphies of various coelurosaurian clades below, and comparisons of the synapomorphies of some clades indicate that many of the features are independently evolved in different coelurosurian clades (node numbers are indicated in Supplementary Fig. S3).

Table S2 | Additional steps required when *Fukuivenator* is placed in alternative systematic positions.

Systematic Positions	Constraints	Steps Required
As a member of Paraves	Force+ [11 12 13 14 15 16 17 18 19 20 21 22 23 24 25 26 27 28 29 30 31 32 33 42 44 50 51 52 53 54 55 56 57 58 59 60 61 62 63 82 83 84 85 86 87 88 89 90 91 92 93 94 95 96 97 98 99 100 101 102 103 104 105 106 107 108 111];	2083
As a sister taxon to Paraves	Force=(111 (11 12 13 14 15 16 17 18 19 20 21 22 23 24 25 26 27 28 29 30 31 32 33 42 44 50 51 52 53 54 55 56 57 58 59 60 61 62 63 82 83 84 85 86 87 88 89 90 91 92 93 94 95 96 97 98 99 100 101 102 103 104 105 106 107 108));	2084
As a sister taxon to Deinonychosauria	Force=(111 (11 12 13 14 15 16 17 18 19 20 21 22 23 24 25 26 27 28 29 30 31 32 33 50 51 52 53 54 55 56 57 58 59 60 61 62 63));	2093
As a member of Deinonychosauria	Force+ [111 11 12 13 14 15 16 17 18 19 20 21 22 23 24 25 26 27 28 29 30 31 32 33 50 51 52 53 54 55 56 57 58 59 60 61 62 63];	2083
As a member of Dromaeosauridae	Force+ [11 12 13 14 15 16 17 18 19 20 21 22 23 24 25 26 27 28 29 30 31 32 33 111];	2083
As a sister taxon to Dromaeosauridae	Force=(111 (11 12 13 14 15 16 17 18 19 20 21 22 23 24 25 26 27 28 29 30 31 32 33));	2087

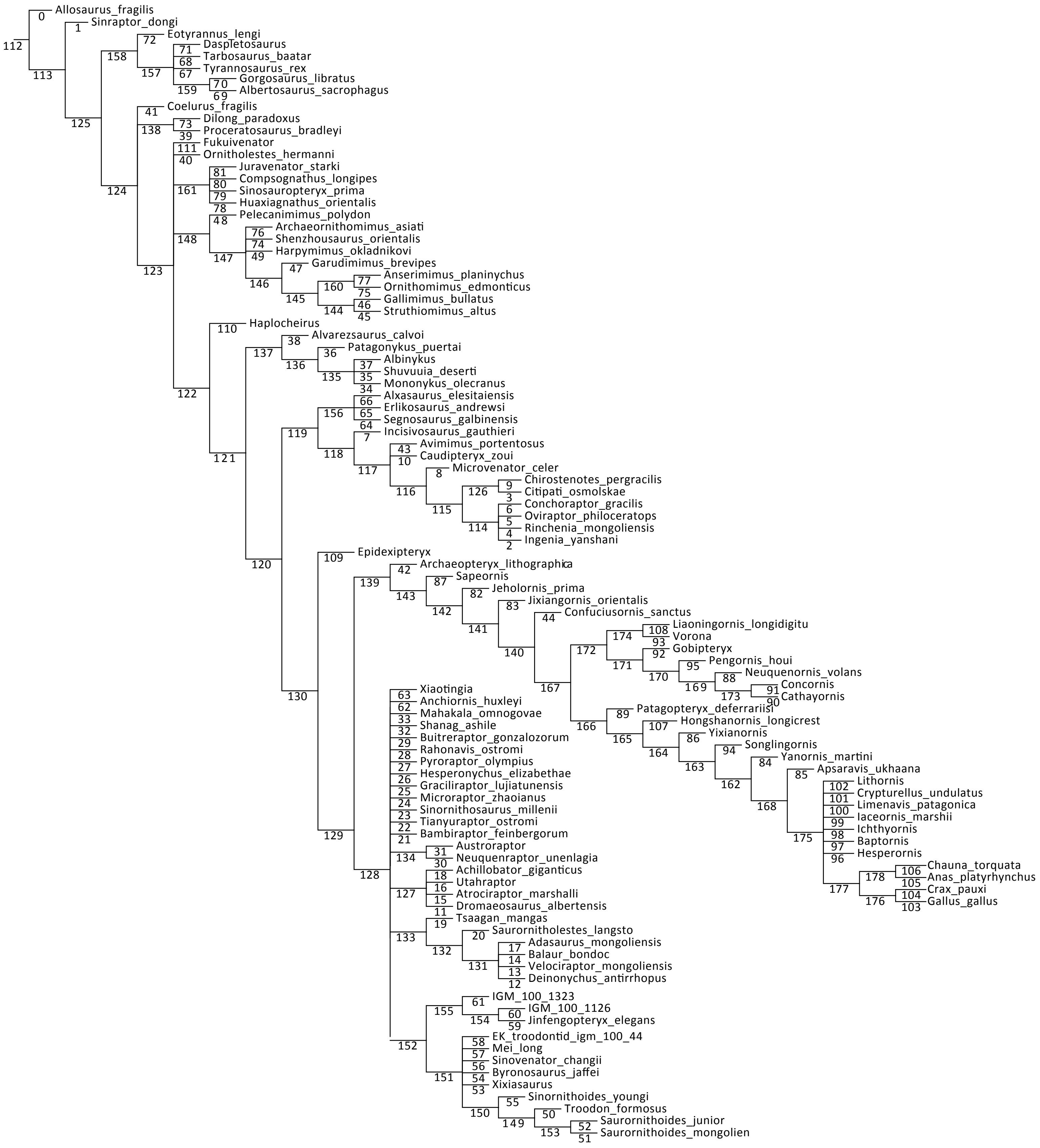

Figure 3 | Strict consensus tree of 100000 most parsimonious trees produced by an analysis on a dataset including *Fukuivenator*.

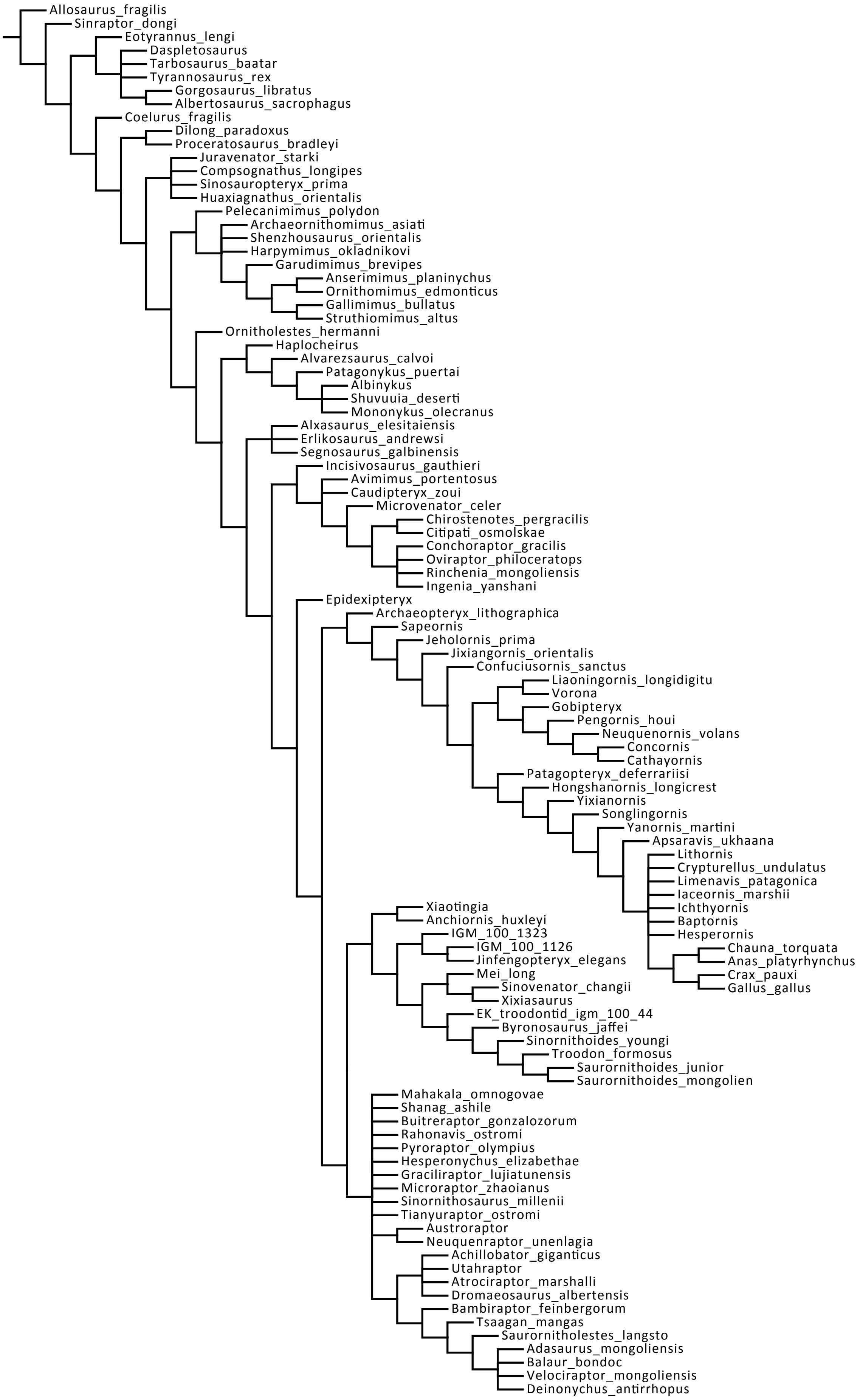

Figure S4 | Strict consensus tree of 100000 most parsimonious trees produced by an analysis on a dataset excluding *Fukuivenator*.

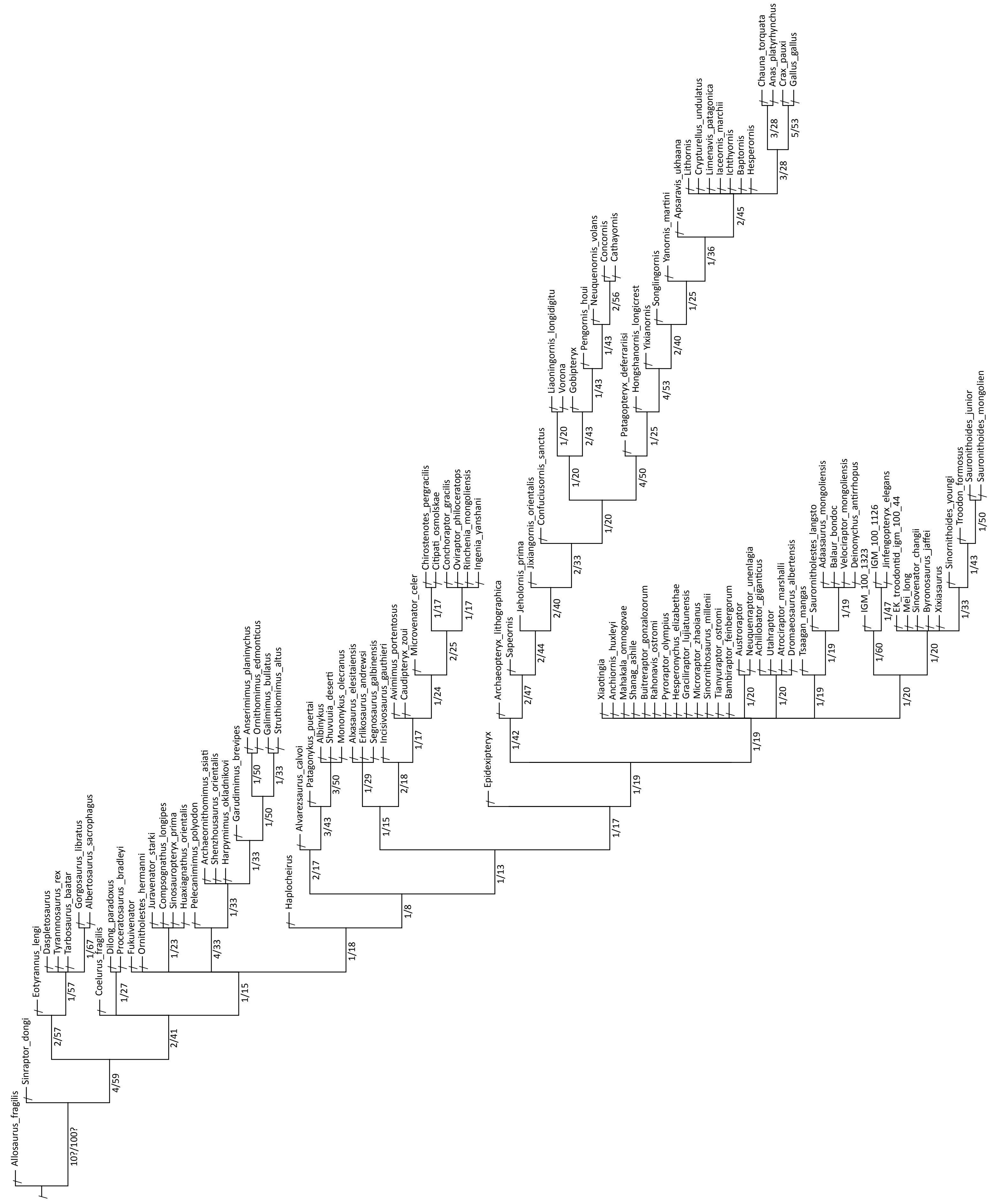

Figure S5 | Bootstrap and Bremer analysises. Values at each node indicate bootstrap and Bremer support, respectively.

List of synapomorphies for various coelurosaurian clades:

Allosaurus_fragilis : All trees:
No autapomorphies:
Sinraptor_dongi : All trees:
Char. 243: 0 --> 1
Char. 249: 0 --> 1
Char. 410: 0 --> 1
Ingenia_yanshani : Some trees:
Char. 197: 0 --> 1
Citipati_osmolskae : All trees:
Char. 15: 1 --> 2
Char. 22: 1 --> 0
Char. 183: 0 --> 2
Char. 280: 0 --> 1
Rinchenia_mongoliensis : All trees:
Char. 273: 0 --> 1
Some trees:
Char. 260: 0 --> 1
Char. 272: 0 --> 1
Oviraptor_philoceratops : Some trees:
Char. 257: 0 --> 1
Conchoraptor_gracilis : Some trees:
Char. 109: 1 --> 2
Char. 167: 1 --> 0
Char. 202: 0 --> 1
Char. 278: 1 --> 0
Char. 295: 0 --> 1
Incisivosaurus_gauthieri : All trees:
Char. 18: 0 --> 1
Char. 38: 0 --> 1
Char. 40: 1 --> 0
Char. 50: 0 --> 1
Char. 53: 0 --> 1
Char. 72: 0 --> 1
Char. 244: 0 --> 1
Char. 255: 0 --> 1

Char. 300: 0 --> 1
Char. 303: 0 --> 1
Some trees:
Char. 35: 0 --> 1
Char. 52: 1 --> 0
Char. 245: 1 --> 0
Microvenator_celer : All trees:
Char. 101: 1 --> 0
Char. 135: 1 --> 0
Char. 161: 1 --> 0
Char. 163: 1 --> 0
Char. 187: 0 --> 1
Some trees:
Char. 99: 0 --> 1
Chirostenotes_pergracilis : All trees:
Char. 26: 1 --> 0
Char. 54: 0 --> 1
Char. 133: 0 --> 1
Char. 166: 0 --> 2
Char. 187: 0 --> 1
Char. 202: 0 --> 2
Char. 467: 0 --> 1
Some trees:
Char. 99: 0 --> 1
Caudipteryx_zoui : All trees:
Char. 202: 0 --> 1
Char. 350: 0 --> 1
Char. 437: 1 --> 2
Char. 466: 0 --> 1
Some trees:
Char. 34: 0 --> 1
Char. 183: 0 --> 1
Char. 259: 1 --> 0
Dromaeosaurus_albertensis : All trees:
Char. 246: 1 --> 0
Deinonychus_antirrhopus : All trees:
Char. 39: 2 --> 1

Char. 177: 1 --> 0
Char. 247: 0 --> 1
Some trees:
Char. 134: 1 --> 0
Char. 197: 1 --> 0
Char. 198: 1 --> 0
Velociraptor_mongoliensis : All trees:
Char. 228: 0 --> 1
Char. 234: 0 --> 1
Char. 264: 2 --> 1
Balaur_bondoc : All trees:
Char. 146: 0 --> 1
Char. 156: 2 --> 0
Char. 172: 1 --> 0
Char. 178: 1 --> 2
Char. 197: 1 --> 2
Char. 199: 0 --> 1
Char. 200: 1 --> 0
Char. 258: 0 --> 1
Char. 317: 0 --> 2
Char. 389: 0 --> 2
Char. 390: 0 --> 1
Char. 401: 0 --> 2
Char. 404: 0 --> 1
Char. 457: 1 --> 0
Atrociraptor_marshalli : Some trees:
Char. 247: 0 --> 1
Char. 248: 0 --> 1
Char. 259: 0 --> 1
Utahraptor : Some trees:
Char. 34: 1 --> 0
Char. 201: 1 --> 0
Adasaurus_mongoliensis : All trees:
Char. 316: 0 --> 1
Char. 434: 0 --> 2
Some trees:
Char. 186: 1 --> 0

Char. 237: 0 --> 1
Achillobator_giganticus : Some trees:
Char. 157: 1 --> 0
Char. 160: 0 --> 1
Tsaagan_mangas : All trees:
Char. 27: 1 --> 0
Char. 53: 0 --> 1
Char. 57: 1 --> 0
Char. 105: 1 --> 0
Some trees:
Char. 237: 0 --> 1
Saurornitholestes_langsto : All trees:
Char. 167: 2 --> 0
Bambiraptor_feinbergorum : All trees:
Char. 247: 0 --> 1
Some trees:
Char. 94: 0 --> 1
Char. 228: 0 --> 1
Char. 243: 0 --> 1
Char. 301: 0 --> 1
Char. 317: 0 --> 1
Tianyuraptor_ostromi : Some trees:
Char. 105: 1 --> 0
Char. 132: 1 --> 0
Char. 162: 0 --> 1
Char. 166: 0 --> 2
Char. 179: 2 --> 0
Char. 404: 0 --> 1
Char. 411: 0 --> 1
Char. 468: 1 --> 0
Char. 469: 1 --> 0
Sinornithosaurus_millenii :
Some trees:
Char. 153: 1 --> 0
Char. 157: 1 --> 0
Char. 195: 0 --> 1
Char. 212: 0 --> 1

Char. 227: 0 --> 1
Char. 302: 0 --> 1
Char. 460: 1 --> 0
Microraptor_zhaoianus : Some trees:
Char. 85: 1 --> 0
Char. 87: 1 --> 0
Char. 101: 1 --> 0
Char. 134: 0 --> 1
Char. 197: 0 --> 1
Char. 198: 0 --> 1
Char. 204: 0 --> 1
Char. 207: 0 --> 1
Char. 334: 0 --> 1
Char. 355: 0 --> 1
Graciliraptor_lujiatunensis : Some trees:
Char. 82: 1 --> 2
Char. 142: 0 --> 1
Hesperonychus_elizabethae : Some trees:
Char. 180: 0 --> 1
Pyroraptor_olympius : All trees:
No autapomorphies:
Rahonavis_ostromi : All trees:
Char. 234: 0 --> 1
Char. 442: 0 --> 1
Some trees:
Char. 111: 1 --> 0
Char. 117: 0 --> 1
Char. 142: 0 --> 1
Char. 173: 1 --> 2
Char. 180: 0 --> 1
Char. 183: 1 --> 2
Char. 190: 0 --> 1
Char. 206: 0 --> 1
Char. 231: 0 --> 1
Char. 235: 0 --> 1
Char. 317: 0 --> 1
Char. 417: 0 --> 1

Buitreraptor_gonzalozorum : Some trees:
Char. 41: 0 --> 1
Char. 95: 0 --> 1
Char. 112: 1 --> 0
Char. 193: 0 --> 1
Char. 373: 0 --> 1
Neuquenraptor_unenlagia : All trees:
No autapomorphies:
Austroraptor : All trees:
Char. 140: 1 --> 0
Shanag_ashile : Some trees:
Char. 19: 2 --> 1
Char. 28: 1 --> 0
Char. 70: 0 --> 1
Char. 82: 2 --> 1
Char. 88: 0 --> 1
Char. 236: 0 --> 1
Char. 238: 0 --> 1
Mahakala_omnogovae : Some trees:
Char. 109: 0 --> 1
Char. 163: 1 --> 2
Char. 296: 0 --> 1
Char. 352: 1 --> 0
Char. 362: 1 --> 0
Char. 410: 0 --> 1
Mononykus_olecranus : Some trees:
Char. 267: 0 --> 1
Char. 352: 0 --> 1
Char. 363: 1 --> 2
Shuvuuia_deserti : Some trees:
Char. 357: 0 --> 1
Patagonykus_puertai : All trees:
Char. 412: 0 --> 1
Albinykus : All trees:
Char. 199: 0 --> 1
Char. 419: 0 --> 1
Char. 424: 0 --> 1

Alvarezsaurus_calvoi : All trees:
Char. 264: 0 --> 12
Char. 317: 0 --> 2
Proceratosaurus_bradleyi : All trees:
Char. 242: 0 --> 1
Ornitholestes_hermanni : All trees:
Char. 122: 0 --> 1
Some trees:
Char. 34: 0 --> 1
Char. 52: 0 --> 1
Char. 55: 0 --> 1
Char. 80: 0 --> 1
Char. 101: 0 --> 1
Char. 118: 0 --> 1
Char. 176: 0 --> 1
Char. 232: 1 --> 0
Char. 261: 0 --> 2
Char. 264: 0 --> 1
Char. 450: 1 --> 0
Char. 464: 0 --> 1
Coelurus_fragilis : All trees:
Char. 143: 0 --> 1
Some trees:
Char. 195: 0 --> 1
Archaeopteryx_lithographi : All trees:
Char. 59: 0 --> 1
Char. 122: 02 --> 1
Char. 123: 1 --> 0
Char. 134: 0 --> 1
Char. 139: 0 --> 1
Char. 152: 1 --> 0
Char. 168: 2 --> 3
Char. 173: 1 --> 2
Char. 197: 1 --> 0
Char. 231: 0 --> 1
Char. 386: 1 --> 0
Some trees:

Char. 28: 0 --> 1
Char. 38: 0 --> 1
Char. 75: 1 --> 2
Avimimus_portentosus : All trees:
Char. 11: 0 --> 1
Char. 15: 1 --> 0
Char. 95: 1 --> 0
Char. 97: 1 --> 0
Char. 173: 1 --> 0
Char. 184: 1 --> 0
Char. 197: 0 --> 1
Char. 198: 0 --> 1
Char. 199: 0 --> 1
Char. 202: 0 --> 2
Char. 268: 0 --> 1
Char. 389: 0 --> 2
Char. 417: 0 --> 1
Some trees:
Char. 35: 0 --> 1
Char. 99: 0 --> 1
Confuciusornis_sanctus : All trees:
Char. 22: 1 --> 0
Char. 31: 1 --> 0
Char. 67: 0 --> 1
Char. 73: 0 --> 1
Char. 102: 0 --> 1
Char. 134: 0 --> 1
Char. 152: 1 --> 0
Char. 194: 0 --> 1
Char. 195: 0 --> 2
Char. 196: 1 --> 0
Char. 219: 1 --> 2
Char. 231: 0 --> 1
Char. 302: 0 --> 1
Char. 307: 0 --> 1
Char. 309: 0 --> 1
Char. 350: 1 --> 0

Char. 351: 1 --> 0
Char. 363: 1 --> 2
Char. 365: 0 --> 1
Char. 444: 1 --> 0
Some trees:
Char. 303: 0 --> 12
Struthiomimus_altus : All trees:
Char. 67: 0 --> 1
Char. 178: 1 --> 0
Char. 354: 0 --> 1
Char. 386: 1 --> 0
Char. 437: 1 --> 2
Gallimimus_bullatus : All trees:
Char. 427: 0 --> 1
Some trees:
Char. 352: 0 --> 1
Garudimimus_brevipes : All trees:
Char. 245: 1 --> 0
Char. 421: 0 --> 1
Char. 434: 0 --> 3
Char. 463: 0 --> 1
Some trees:
Char. 298: 0 --> 1
Char. 300: 0 --> 1
Pelecanimimus_polydon : All trees:
Char. 83: 0 --> 1
Char. 84: 0 --> 1
Char. 148: 0 --> 1
Some trees:
Char. 87: 1 --> 0
Harpymimus_okladnikovi : All trees:
Char. 264: 0 --> 1
Some trees:
Char. 212: 1 --> 0
Char. 383: 0 --> 1
Troodon_formosus : All trees:
Char. 53: 1 --> 0

Char. 197: 1 --> 0
Char. 224: 1 --> 0
Saurornithoides_mongoliensis : All trees:
No autapomorphies:
Saurornithoides_junior : All trees:
Char. 421: 0 --> 1
Char. 458: 0 --> 1
Some trees:
Char. 248: 1 --> 0
Xixiasaurus : Some trees:
Char. 19: 1 --> 0
Char. 22: 1 --> 0
Char. 83: 1 --> 0
Byronosaurus_jaffei : Some trees:
Char. 248: 1 --> 0
Sinornithoides_youngi : All trees:
Char. 118: 2 --> 1
Char. 139: 0 --> 1
Char. 202: 2 --> 1
Char. 302: 0 --> 1
Sinovenator_changii : All trees:
Char. 82: 2 --> 1
Char. 243: 0 --> 1
Char. 247: 0 --> 1
Some trees:
Char. 19: 1 --> 0
Char. 99: 0 --> 1
Char. 101: 1 --> 0
Char. 110: 1 --> 0
Char. 404: 0 --> 1
Char. 411: 0 --> 1
Char. 455: 1 --> 0
Mei_long : All trees:
Char. 31: 0 --> 1
Char. 44: 1 --> 0
Char. 102: 0 --> 1
Char. 197: 1 --> 0

Char. 410: 0 --> 1
Char. 434: 0 --> 1
Some trees:
Char. 38: 2 --> 1
EK_troodontid_igm_100_44 : All trees:
Char. 152: 1 --> 0
Jinfengopteryx_elegans : All trees:
Char. 44: 1 --> 0
Char. 115: 1 --> 2
IGM_100_1126 : All trees:
Char. 176: 2 --> 1
IGM_100_1323 : Some trees:
Char. 22: 1 --> 0
Anchiornis_huxleyi : All trees:
Char. 134: 0 --> 1
Char. 152: 1 --> 0
Char. 153: 01 --> 1
Char. 175: 0 --> 1
Char. 211: 0 --> 1
Char. 265: 1 --> 2
Char. 390: 0 --> 1
Some trees:
Char. 140: 1 --> 0
Char. 200: 1 --> 0
Char. 233: 0 --> 1
Xiaotingia : All trees:
Char. 203: 1 --> 0
Char. 442: 0 --> 1
Some trees:
Char. 154: 0 --> 1
Char. 166: 0 --> 1
Char. 264: 0 --> 2
Char. 391: 0 --> 1
Char. 396: 0 --> 1
Char. 437: 0 --> 2
Segnosaurus_galbinensis : Some trees:
Char. 109: 0 --> 1

Char. 150: 0 --> 1
Char. 164: 0 --> 1
Char. 437: 01 --> 2
Erlikosaurus_andrewsi : All trees:
No autapomorphies:
Alxasaurus_elesitaiensis : All trees:
No autapomorphies:
Tyrannosaurus_rex : Some trees:
Char. 8: 0 --> 1
Char. 187: 0 --> 1
Char. 234: 0 --> 1
Tarbosaurus_baatar : All trees:
No autapomorphies:
Albertosaurus_sacrophagus : All trees:
No autapomorphies:
Gorgosaurus_libratus : All trees:
No autapomorphies:
Daspletosaurus : All trees:
No autapomorphies:
Eotyrannus_lengi : All trees:
Char. 37: 1 --> 0
Dilong_paradoxus : All trees:
Char. 253: 0 --> 1
Char. 254: 0 --> 1
Char. 298: 0 --> 1
Some trees:
Char. 261: 2 --> 0
Shenzhousaurus_orientalis : All trees:
Char. 242: 0 --> 1
Some trees:
Char. 157: 0 --> 1
Ornithomimus_edmonticus : All trees:
No autapomorphies:
Archaeornithomimus_asiati : All trees:
Char. 159: 0 --> 1
Char. 165: 0 --> 1
Char. 167: 0 --> 1

Char. 168: 1 --> 0
Char. 180: 0 --> 1
Char. 186: 0 --> 1
Char. 191: 0 --> 1
Char. 195: 0 --> 1
Char. 196: 1 --> 0
Char. 209: 0 --> 1
Some trees:
Char. 93: 1 --> 0
Char. 109: 0 --> 1
Char. 161: 1 --> 0
Char. 178: 1 --> 0
Char. 181: 1 --> 0
Char. 352: 0 --> 1
Anserimimus_planinychus : All trees:
Char. 390: 0 --> 1
Huaxiagnathus_orientalis : All trees:
Char. 120: 01 --> 2
Some trees:
Char. 474: 0 --> 1
Sinosauropteryx_prima : All trees:
Char. 232: 1 --> 0
Some trees:
Char. 117: 0 --> 1
Char. 141: 0 --> 1
Char. 390: 1 --> 0
Compsognathus_longipes : All trees:
Char. 98: 1 --> 0
Some trees:
Char. 45: 0 --> 1
Char. 141: 0 --> 1
Char. 390: 1 --> 0
Juravenator_starki : All trees:
Char. 197: 0 --> 1
Char. 266: 0 --> 1
Some trees:
Char. 22: 1 --> 0

Char. 39: 1 --> 2
Char. 40: 0 --> 1
Char. 156: 12 --> 0
Jeholornis_prima : All trees:
Char. 117: 0 --> 1
Char. 163: 2 --> 0
Char. 197: 12 --> 0
Char. 243: 0 --> 1
Char. 269: 0 --> 1
Jixiangornis_orientalis : All trees:
Char. 144: 1 --> 0
Char. 153: 0 --> 1
Char. 158: 1 --> 0
Char. 173: 1 --> 0
Char. 240: 0 --> 1
Char. 321: 0 --> 1
Char. 324: 0 --> 1
Char. 352: 0 --> 1
Char. 389: 1 --> 0
Char. 410: 0 --> 1
Char. 425: 1 --> 0
Char. 431: 1 --> 0
Yanornis_martini : All trees:
Char. 193: 1 --> 0
Char. 201: 1 --> 0
Char. 204: 1 --> 3
Char. 468: 0 --> 1
Apsaravis_ukhaana : All trees:
Char. 67: 0 --> 2
Char. 94: 0 --> 1
Char. 138: 1 --> 0
Char. 165: 1 --> 0
Char. 219: 0 --> 2
Char. 269: 0 --> 1
Char. 317: 1 --> 0
Char. 339: 1 --> 0
Char. 341: 1 --> 0

Char. 344: 1 --> 0
Char. 347: 1 --> 0
Char. 349: 1 --> 0
Char. 371: 0 --> 1
Char. 372: 0 --> 1
Char. 373: 0 --> 1
Char. 374: 0 --> 1
Char. 394: 1 --> 0
Char. 421: 1 --> 2
Char. 422: 0 --> 1
Char. 423: 0 --> 1
Some trees:
Char. 354: 0 --> 1
Yixianornis : All trees:
Char. 70: 1 --> 0
Char. 87: 0 --> 1
Char. 118: 1 --> 0
Char. 142: 1 --> 0
Char. 144: 1 --> 0
Char. 168: 0 --> 3
Char. 173: 12 --> 0
Char. 185: 0 --> 1
Char. 232: 1 --> 0
Char. 234: 1 --> 0
Sapeornis : All trees:
Char. 73: 0 --> 1
Char. 118: 1 --> 0
Char. 120: 2 --> 3
Char. 131: 0 --> 2
Char. 140: 0 --> 1
Char. 161: 1 --> 0
Char. 174: 1 --> 0
Char. 322: 0 --> 1
Char. 365: 0 --> 1
Char. 370: 0 --> 1
Char. 390: 0 --> 1
Neuquenornis_volans : All trees:

Char. 131: 2 --> 1
Char. 264: 2 --> 0
Char. 265: 2 --> 1
Char. 317: 1 --> 0
Char. 444: 1 --> 0
Patagopteryx_deferrariisi : All trees:
Char. 264: 2 --> 0
Char. 297: 0 --> 1
Char. 317: 1 --> 0
Char. 329: 1 --> 0
Char. 348: 0 --> 1
Char. 363: 1 --> 0
Char. 376: 0 --> 1
Char. 379: 1 --> 0
Char. 384: 1 --> 0
Char. 412: 0 --> 1
Char. 431: 1 --> 0
Cathayornis : All trees:
Char. 177: 2 --> 1
Concornis : All trees:
Char. 168: 0 --> 1
Gobipteryx : All trees:
Char. 197: 2 --> 1
Char. 269: 0 --> 1
Vorona :
All trees:
Char. 197: 2 --> 1
Char. 426: 1 --> 0
Songlingornis : All trees:
Char. 332: 1 --> 0
Pengornis_houi : All trees:
Char. 139: 0 --> 1
Char. 154: 1 --> 0
Char. 272: 1 --> 0
Char. 354: 0 --> 1
Char. 355: 1 --> 2
Char. 356: 0 --> 1

Char. 357: 0 --> 1
Char. 398: 0 --> 1
Char. 469: 1 --> 0
Char. 471: 0 --> 1
Hesperornis : Some trees:
Char. 301: 1 --> 0
Char. 329: 1 --> 0
Char. 330: 2 --> 1
Char. 433: 0 --> 2
Baptornis : Some trees:
Char. 199: 3 --> 2
Ichthyornis : All trees:
Char. 353: 0 --> 1
Char. 388: 1 --> 2
Char. 400: 0 --> 1
Some trees:
Char. 40: 1 --> 0
Char. 43: 0 --> 1
Char. 87: 0 --> 1
Char. 92: 0 --> 1
Char. 111: 1 --> 0
Char. 119: 2 --> 3
Char. 124: 1 --> 0
Char. 128: 1 --> 0
Char. 154: 0 --> 1
Char. 189: 0 --> 1
Char. 292: 0 --> 1
Char. 303: 0 --> 2
Char. 329: 1 --> 2
Char. 382: 0 --> 1
Char. 448: 1 --> 0
Laceornis_marshii : All trees:
Char. 336: 0 --> 1
Char. 337: 0 --> 1
Some trees:
Char. 189: 0 --> 1
Char. 329: 1 --> 2

Char. 331: 0 --> 1
Char. 354: 0 --> 1
Char. 391: 23 --> 4
Char. 395: 0 --> 1
Char. 419: 1 --> 2
Limenavis_patagonica : All trees:
No autapomorphies:
Crypturellus_undulatus : All trees:
Char. 138: 1 --> 0
Char. 166: 0 --> 2
Char. 299: 0 --> 1
Char. 345: 1 --> 0
Char. 376: 0 --> 1
Char. 388: 1 --> 0
Some trees:
Char. 48: 0 --> 1
Char. 50: 0 --> 1
Char. 73: 0 --> 1
Char. 92: 0 --> 1
Char. 191: 1 --> 0
Char. 215: 1 --> 0
Char. 296: 1 --> 0
Char. 306: 1 --> 0
Char. 319: 0 --> 1
Char. 338: 0 --> 2
Char. 359: 0 --> 1
Char. 474: 1 --> 0
Lithornis : Some trees:
Char. 130: 1 --> 0
Char. 317: 0 --> 2
Char. 329: 1 --> 2
Char. 354: 0 --> 1
Char. 391: 234 --> 3
Char. 395: 01 --> 0
Char. 419: 12 --> 1
Char. 421: 1 --> 0
Gallus_gallus : All trees:

Char. 20: 1 --> 0
Char. 65: 0 --> 1
Char. 107: 1 --> 0
Char. 138: 1 --> 0
Char. 158: 0 --> 1
Char. 217: 1 --> 0
Char. 395: 1 --> 0
Char. 469: 1 --> 0
Char. 470: 0 --> 1
Char. 472: 0 --> 1
Char. 474: 1 --> 0
Some trees:
Char. 140: 1 --> 0
Char. 405: 1 --> 2
Crax_pauxi : All trees:
Char. 15: 12 --> 0
Char. 93: 1 --> 0
Char. 130: 1 --> 0
Char. 179: 2 --> 0
Char. 437: 1 --> 0
Some trees:
Char. 155: 1 --> 0
Anas_platyrhynchus : All trees:
Char. 22: 1 --> 0
Char. 23: 0 --> 1
Char. 64: 0 --> 2
Char. 70: 0 --> 1
Char. 132: 0 --> 1
Char. 157: 1 --> 0
Char. 179: 2 --> 1
Char. 200: 0 --> 1
Char. 213: 0 --> 1
Char. 287: 1 --> 2
Char. 332: 1 --> 2
Char. 342: 1 --> 0
Char. 355: 1 --> 0
Char. 382: 0 --> 1

Char. 396: 1 --> 0
Char. 413: 2 --> 1
Char. 417: 2 --> 1
Char. 437: 1 --> 2
Chauna_torquata : All trees:
Char. 0: 1 --> 0
Char. 15: 12 --> 0
Char. 96: 1 --> 0
Char. 99: 2 --> 1
Char. 124: 2 --> 1
Char. 128: 1 --> 0
Char. 130: 1 --> 0
Char. 148: 0 --> 1
Char. 208: 1 --> 0
Char. 264: 1 --> 2
Char. 317: 0 --> 2
Char. 330: 3 --> 4
Char. 331: 1 --> 0
Char. 347: 1 --> 0
Char. 353: 0 --> 1
Char. 355: 1 --> 2
Char. 421: 1 --> 2
Char. 437: 1 --> 0
Char. 454: 0 --> 1
Char. 471: 0 --> 1
Some trees:
Char. 169: 1 --> 0
Hongshanornis_longicrest : All trees:
Char. 83: 0 --> 1
Char. 150: 03 --> 2
Char. 204: 1 --> 0
Char. 340: 0 --> 1
Char. 364: 1 --> 0
Char. 368: 1 --> 2
Char. 437: 1 --> 2
Liaoningornis_longidigitu : All trees:
Char. 420: 1 --> 0

Char. 427: 0 --> 1
Char. 429: 0 --> 2
Char. 430: 1 --> 0
Epidexipteryx : All trees:
Char. 2: 1 --> 0
Char. 87: 0 --> 1
Char. 114: 0 --> 1
Char. 166: 0 --> 2
Char. 176: 2 --> 0
Char. 177: 1 --> 2
Char. 204: 0 --> 1
Char. 219: 0 --> 1
Char. 247: 0 --> 1
Char. 426: 0 --> 1
Some trees:
Char. 80: 0 --> 1
Haplocheirus : All trees:
Char. 2: 1 --> 0
Char. 4: 0 --> 1
Char. 23: 0 --> 1
Char. 29: 0 --> 1
Char. 38: 0 --> 2
Char. 46: 0 --> 1
Char. 70: 0 --> 1
Char. 72: 0 --> 1
Char. 95: 1 --> 0
Char. 105: 1 --> 0
Char. 132: 0 --> 1
Char. 136: 0 --> 1
Char. 166: 0 --> 1
Char. 242: 0 --> 1
Char. 245: 1 --> 0
Char. 338: 2 --> 0
Char. 361: 0 --> 1
Char. 362: 1 --> 2
Char. 465: 0 --> 1
Some trees:

Char. 28: 0 --> 1
Char. 34: 0 --> 1
Char. 39: 1 --> 0
Char. 57: 0 --> 1
Char. 82: 2 --> 1
Char. 98: 1 --> 0
Char. 161: 1 --> 0
Char. 181: 1 --> 0
Char. 211: 0 --> 1
Char. 298: 0 --> 1
Fukuivenator : All trees:
Char. 41: 0 --> 1
Char. 48: 0 --> 1
Char. 54: 0 --> 1
Char. 108: 0 --> 1
Char. 109: 0 --> 1
Char. 117: 0 --> 1
Char. 138: 0 --> 1
Char. 228: 0 --> 1
Char. 236: 0 --> 1
Char. 238: 0 --> 1
Char. 264: 01 --> 2
Char. 316: 1 --> 0
Char. 370: 0 --> 1
Char. 374: 0 --> 1
Char. 375: 0 --> 1
Char. 417: 0 --> 2
Char. 433: 0 --> 1
Char. 434: 0 --> 3
Char. 437: 0 --> 1
Char. 454: 0 --> 1
Char. 462: 0 --> 1
Some trees:
Char. 9: 0 --> 1
Char. 19: 0 --> 2
Char. 24: 1 --> 0
Char. 43: 0 --> 1

Char. 87: 1 --> 0
Char. 91: 0 --> 1
Char. 98: 1 --> 0
Char. 99: 0 --> 1
Char. 102: 0 --> 1
Char. 118: 01 --> 0

Char. 141: 0 --> 1
Char. 152: 0 --> 1
Char. 181: 1 --> 0
Char. 185: 0 --> 1
Char. 203: 0 --> 1
Char. 242: 0 --> 2
Char. 244: 0 --> 1
Char. 282: 0 --> 1
Char. 363: 0 --> 1
Char. 381: 1 --> 0
Char. 413: 2 --> 0
Char. 448: 0 --> 1
Char. 455: 0 --> 1
Char. 464: 0 --> 1
Node 113 : All trees:
No synapomorphies
Node 114 : All trees:
Char. 183: 0 --> 1
Char. 463: 0 --> 2
Node 115 : All trees:
Char. 112: 0 --> 1
Char. 139: 1 --> 0
Char. 180: 0 --> 1
Char. 269: 0 --> 1
Node 116 : All trees:
Char. 264: 01 --> 2
Char. 317: 0 --> 1
Some trees:
Char. 140: 0 --> 1
Node 117 : All trees:

Char. 21: 0 --> 1
Char. 66: 0 --> 1
Some trees:
Char. 52: 01 --> 1
Char. 67: 1 --> 2
Char. 81: 0 --> 1
Node 118 : All trees:
Char. 8: 2 --> 1
Char. 19: 1 --> 0
Char. 56: 0 --> 1
Char. 77: 0 --> 1
Char. 302: 0 --> 1
Some trees:
Char. 259: 0 --> 1
Node 119 : All trees:
Char. 12: 0 --> 1
Char. 23: 0 --> 1
Char. 63: 0 --> 1
Char. 64: 0 --> 2
Char. 177: 1 --> 0
Some trees:
Char. 118: 1 --> 0
Char. 183: 1 --> 0
Node 120 : All trees:
Char. 8: 0 --> 2
Char. 128: 0 --> 1
Char. 130: 0 --> 1
Char. 141: 1 --> 0
Char. 152: 0 --> 1
Char. 156: 1 --> 2
Char. 165: 0 --> 1
Some trees:
Char. 20: 1 --> 0
Char. 69: 1 --> 0
Char. 102: 1 --> 0
Char. 151: 1 --> 0
Char. 209: 1 --> 0

Char. 464: 0 --> 1
Node 121 : All trees:
Char. 11: 1 --> 0
Char. 39: 01 --> 2
Char. 60: 0 --> 1
Char. 73: 1 --> 0
Char. 145: 0 --> 1
Char. 170: 0 --> 1
Char. 174: 0 --> 1
Char. 176: 0 --> 2
Char. 184: 0 --> 1
Some trees:
Char. 57: 01 --> 0
Char. 215: 1 --> 0
Node 122 : All trees:
Char. 83: 0 --> 1
Char. 183: 0 --> 1
Some trees:
Char. 3: 0 --> 1
Char. 19: 02 --> 1
Char. 55: 0 --> 1
Char. 62: 0 --> 1
Char. 87: 1 --> 0
Char. 90: 1 --> 0
Char. 101: 0 --> 1
Char. 115: 0 --> 1
Char. 160: 1 --> 0
Char. 163: 0 --> 1
Char. 173: 0 --> 1
Char. 217: 0 --> 1
Node 123 : Some trees:
Char. 36: 1 --> 0
Char. 37: 1 --> 0
Char. 94: 0 --> 1
Char. 97: 0 --> 1
Char. 161: 0 --> 1
Char. 244: 1 --> 0

Node 124 : All trees:
Char. 95: 0 --> 1
Char. 177: 0 --> 1
Char. 263: 0 --> 1
Char. 265: 0 --> 1
Some trees:
Char. 1: 1 --> 0
Char. 15: 0 --> 1
Char. 92: 1 --> 0
Char. 104: 0 --> 1
Char. 153: 1 --> 0
Char. 157: 0 --> 1
Char. 237: 1 --> 0
Char. 251: 1 --> 0
Char. 262: 1 --> 0
Char. 450: 0 --> 1
Char. 463: 1 --> 0
Node 125 : All trees:
Char. 24: 0 --> 1
Char. 34: 1 --> 0
Char. 39: 0 --> 1
Char. 40: 1 --> 0
Char. 90: 0 --> 1
Char. 196: 0 --> 1
Char. 232: 0 --> 1
Char. 242: 1 --> 0
Some trees:
Char. 100: 1 --> 0
Char. 195: 1 --> 0
Node 126 : All trees:
Char. 9: 0 --> 1
Char. 112: 1 --> 2
Char. 137: 0 --> 1
Char. 437: 1 --> 0
Node 127 : Some trees:
Char. 27: 1 --> 0
Char. 82: 1 --> 0

Char. 90: 0 --> 1
Char. 176: 2 --> 1
Char. 177: 1 --> 0
Char. 237: 0 --> 1
Node 128 : All trees:
Char. 60: 1 --> 0
Char. 73: 0 --> 1
Char. 74: 0 --> 1
Char. 108: 0 --> 1
Char. 203: 0 --> 1
Char. 383: 0 --> 1
Some trees:
Char. 22: 1 --> 0
Char. 38: 0 --> 2
Char. 70: 0 --> 01
Char. 352: 0 --> 1
Node 129 : All trees:
Char. 132: 0 --> 1
Char. 137: 0 --> 1
Char. 172: 0 --> 1
Some trees:
Char. 179: 0 --> 2
Char. 456: 1 --> 0
Node 130 : All trees:
Char. 83: 1 --> 0
Char. 133: 0 --> 1
Char. 138: 0 --> 1
Char. 197: 0 --> 1
Node 131 : All trees:
Char. 206: 0 --> 1
Some trees:
Char. 109: 0 --> 1
Char. 198: 0 --> 1

Node 132 : All trees:
Char. 59: 0 --> 1
Char. 250: 0 --> 1

Node 133 : All trees:
Char. 18: 0 --> 1
Node 134 : All trees:
Char. 107: 0 --> 1
Some trees:
Char. 197: 01 --> 0
Char. 264: 0 --> 12
Node 135 : All trees:
Char. 190: 0 --> 1
Char. 193: 0 --> 1
Char. 202: 0 --> 3
Some trees:
Char. 103: 1 --> 0
Char. 176: 2 --> 3
Char. 183: 1 --> 2
Char. 186: 0 --> 1
Char. 189: 0 --> 1
Char. 194: 0 --> 1
Char. 195: 0 --> 2
Char. 356: 0 --> 1
Char. 389: 0 --> 3
Char. 417: 0 --> 1
Node 136 : All trees:
Char. 100: 0 --> 1
Char. 197: 0 --> 1
Char. 198: 0 --> 1
Node 137 : All trees:
Char. 113: 0 --> 1
Char. 163: 1 --> 2
Char. 410: 0 --> 1
Some trees:
Char. 111: 1 --> 2
Node 138 : All trees:
Char. 69: 1 --> 0
Char. 73: 1 --> 0
Node 139 : All trees:
Char. 0: 0 --> 1

Char. 17: 1 --> 2
Char. 45: 1 --> 0
Char. 115: 1 --> 2
Char. 154: 0 --> 1
Char. 164: 0 --> 1
Char. 180: 0 --> 1
Char. 204: 0 --> 3
Char. 235: 0 --> 1
Char. 242: 0 --> 1
Char. 265: 1 --> 2
Char. 283: 0 --> 1
Char. 316: 1 --> 0
Char. 425: 0 --> 1
Char. 443: 0 --> 1
Char. 448: 0 --> 1
Some trees:
Char. 52: 1 --> 0
Char. 69: 0 --> 1
Char. 179: 02 --> 2
Char. 282: 0 --> 1
Char. 466: 0 --> 1
Node 140 : All trees:
Char. 69: 1 --> 0
Char. 120: 2 --> 3
Char. 177: 1 --> 2
Char. 305: 0 --> 1
Char. 308: 0 --> 1
Char. 322: 0 --> 1
Char. 364: 0 --> 1
Char. 370: 0 --> 1
Char. 401: 0 --> 1
Char. 417: 0 --> 1
Char. 424: 0 --> 1
Char. 430: 0 --> 1
Node 141 : All trees:
Char. 110: 1 --> 0
Char. 124: 0 --> 1

Char. 127: 0 --> 1
Char. 316: 0 --> 1
Char. 403: 0 --> 1
Node 142 : All trees:
Char. 79: 0 --> 1
Char. 81: 0 --> 1
Char. 135: 1 --> 3
Char. 142: 0 --> 1
Char. 199: 0 --> 1
Char. 272: 0 --> 1
Char. 338: 2 --> 0
Char. 346: 0 --> 1
Char. 350: 0 --> 1
Char. 351: 0 --> 1
Char. 444: 0 --> 1
Node 143 : All trees:
Char. 44: 1 --> 0
Char. 109: 0 --> 12
Char. 119: 2 --> 0
Char. 168: 2 --> 0
Char. 190: 0 --> 1
Char. 193: 0 --> 1
Char. 198: 0 --> 1
Char. 229: 0 --> 1
Char. 264: 0 --> 2
Char. 317: 0 --> 1
Char. 355: 0 --> 1
Char. 389: 0 --> 1
Char. 431: 0 --> 1
Some trees:
Char. 28: 1 --> 0
Node 144 : All trees:
Char. 27: 0 --> 1
Char. 264: 0 --> 2
Node 145 : All trees:
Char. 30: 0 --> 1
Char. 202: 0 --> 2

Char. 254: 0 --> 1
Char. 263: 1 --> 0
Node 146 : All trees:
Char. 219: 1 --> 2
Some trees:
Char. 109: 0 --> 1
Char. 216: 0 --> 1
Node 147 : All trees:
Char. 219: 0 --> 1
Some trees:
Char. 27: 1 --> 0
Char. 79: 0 --> 1
Char. 81: 0 --> 1
Char. 214: 0 --> 1
Node 148 : All trees:
Char. 10: 0 --> 1
Char. 69: 1 --> 0
Char. 150: 0 --> 1
Char. 213: 0 --> 1
Char. 266: 0 --> 1
Some trees:
Char. 19: 02 --> 2
Char. 22: 1 --> 0
Char. 39: 1 --> 0
Char. 211: 0 --> 1
Char. 212: 0 --> 1
Node 149 : All trees:
Char. 64: 0 --> 1
Node 150 : All trees:
Char. 82: 2 --> 1
Node 151 : All trees:
Char. 223: 0 --> 1
Char. 228: 0 --> 1
Char. 298: 0 --> 1
Char. 300: 0 --> 1
Some trees:
Char. 70: 0 --> 1

Char. 88: 0 --> 1
Node 152 : All trees:
Char. 36: 0 --> 2
Char. 83: 0 --> 1
Char. 118: 1 --> 2
Char. 138: 1 --> 0
Char. 207: 0 --> 1
Char. 433: 0 --> 2
Char. 455: 0 --> 1
Some trees:
Char. 50: 0 --> 1
Char. 52: 1 --> 0
Char. 53: 0 --> 1
Char. 84: 0 --> 1
Char. 202: 0 --> 2
Char. 437: 0 --> 12
Char. 454: 0 --> 1
Node 153 : All trees:
Char. 27: 1 --> 0
Node 154 : All trees:
Char. 243: 0 --> 1
Some trees:
Char. 22: 0 --> 1
Node 155 : All trees:
Char. 26: 1 --> 2
Char. 27: 1 --> 0
Char. 172: 1 --> 0
Char. 180: 0 --> 1
Char. 261: 0 --> 3
Char. 427: 0 --> 1
Some trees:
Char. 28: 1 --> 0
Char. 38: 2 --> 1
Node 156 : All trees:
Char. 68: 0 --> 1
Some trees:
Char. 82: 2 --> 0

Char. 153: 0 --> 2
Char. 169: 0 --> 1
Char. 204: 0 --> 2
Char. 205: 0 --> 1
Node 157 : All trees:
Char. 260: 0 --> 1
Some trees:
Char. 298: 0 --> 1
Char. 300: 0 --> 1
Char. 341: 0 --> 1
Char. 354: 0 --> 1
Char. 356: 0 --> 1
Node 158 : All trees:
Char. 252: 0 --> 1
Char. 253: 0 --> 1
Char. 259: 0 --> 1
Char. 266: 0 --> 1
Node 159 : All trees:
Char. 251: 1 --> 0
Some trees:
Char. 58: 1 --> 0
Node 160 : All trees:
Char. 150: 1 --> 2
Node 161 : Some trees:
Char. 90: 1 --> 0
Char. 94: 1 --> 0
Char. 121: 0 --> 1
Char. 125: 0 --> 1
Char. 208: 0 --> 1
Char. 209: 0 --> 1
Node 162 : All trees:
Char. 83: 0 --> 1
Node 163 : All trees:
Char. 81: 1 --> 0
Char. 219: 1 --> 0
Node 164 : All trees:
Char. 69: 0 --> 1

Char. 79: 1 --> 0
Char. 154: 1 --> 0
Char. 155: 1 --> 0
Char. 344: 0 --> 1
Char. 347: 0 --> 1
Char. 390: 0 --> 1
Char. 427: 0 --> 1
Node 165 : All trees:
Char. 339: 0 --> 1
Char. 356: 0 --> 1
Char. 398: 0 --> 1
Char. 421: 0 --> 1
Node 166 : All trees:
Char. 44: 0 --> 1
Char. 199: 12 --> 3
Char. 204: 3 --> 1
Char. 352: 0 --> 1
Char. 401: 1 --> 2
Char. 403: 1 --> 0
Char. 413: 1 --> 2
Char. 429: 0 --> 1
Node 167 : All trees:
Char. 70: 0 --> 1
Char. 332: 0 --> 1
Char. 334: 0 --> 1
Char. 358: 0 --> 1
Char. 361: 0 --> 1
Char. 389: 1 --> 2
Char. 393: 0 --> 1
Char. 418: 0 --> 1
Node 168 : All trees:
Char. 100: 0 --> 2
Char. 109: 4 --> 5
Char. 212: 0 --> 2
Char. 411: 0 --> 1
Char. 412: 0 --> 1
Char. 428: 0 --> 1

Some trees:
Char. 446: 0 --> 1
Node 169 : All trees:
Char. 315: 0 --> 1
Char. 340: 0 --> 1
Node 170 : All trees:
Char. 79: 1 --> 0
Char. 81: 1 --> 0
Node 171 : All trees:
Char. 433: 0 --> 1
Char. 434: 0 --> 1
Node 172 : All trees:
Char. 385: 0 --> 2
Char. 423: 0 --> 1
Node 173 : All trees:
Char. 326: 1 --> 0
Char. 445: 0 --> 1
Node 174 : All trees:
Char. 422: 0 --> 1
Node 175 : All trees:
Char. 101: 0 --> 1
Char. 132: 1 --> 0
Char. 375: 0 --> 1
Char. 377: 0 --> 1
Char. 387: 0 --> 1
Char. 419: 0 --> 12
Char. 430: 1 --> 2
Char. 432: 0 --> 1
Node 176 : All trees:
Char. 112: 1 --> 2
Char. 131: 01 --> 2
Char. 139: 1 --> 2
Char. 397: 1 --> 2
Char. 429: 2 --> 3
Char. 436: 0 --> 1
Some trees:
Char. 319: 0 --> 1

Char. 338: 0 --> 2

Char. 359: 0 --> 1

Node 177 : All trees:

Char. 276: 0 --> 1

Char. 282: 1 --> 2

Char. 283: 1 --> 2

Char. 287: 0 --> 1

Char. 292: 0 --> 1

Char. 293: 0 --> 1

Char. 311: 0 --> 1

Char. 343: 0 --> 1

Char. 378: 0 --> 1

Char. 402: 0 --> 1

Char. 406: 0 --> 1

Some trees:

Char. 280: 0 --> 1

Char. 289: 0 --> 1

Node 178 : All trees:

Char. 18: 0 --> 1

Char. 53: 0 --> 1

Char. 264: 0 --> 1

Char. 291: 1 --> 2

Char. 307: 0 --> 1

Char. 330: 2 --> 3

Char. 337: 0 --> 1

Char. 388: 1 --> 2

Some trees:

Char. 36: 2 --> 0

Matrix:

Allosaurus_fragilis
?11000?00000001000110010001000001110110010-?000000000010001000000000001000
1000000000001010100100000001001010000000000001000000?01????000000000000000
0?000001000000100000001000000000000?1000001100000010000000000000000000100
000000000-0-000-0001000010000000010000100002101000000000002000000000-0000--

000000000000---00000000?0000[01]0-0????????0000020-00-000-010001000?0-0010000
-0000000000000?0???10-01000--0000000-000?000002000000000000?00000?00-0000?0
000002000?00001-0??001000000[01]0?0000
Sinraptor_dongi
?11000??00?00010001000000010?0000010110010-00?00000000000010000?00000100?
10?000000000101010010000000100101000000000000??????????1?10?0?0??00??0?????
??000010000?010??00001000000000?001000000?000000100000000000000?000?000?00
?000000-0-000-00010000111100010???????0002101???00000000000?000000-0000--000
0000?00000--000000001[01]000???00200??00?????2????????????0010???????????????
????????????????????1????-?0??000-?00000100200000000000000000?00-00??0-??000
2000???001?0??0010000?????????
Ingenia_yanshani
?01?0?????????????????1?1?????1????????????????????????????????????21120-01000?01
11?1---------??????????????????1???01-???2?00???0011120011??00000??100000010002
1?????01012010011010?11101101?0000?00011?000000000??0000??11?2000?000-0-0
01000??????????---?-????00001?0??2100?1000000??1??????0-0??0--0000?000?1?10--?0
00??0??1??0[01]0-?02??????0101??0??0-??0?0?0001000?????1100???00?0???????????
??????00???-0?00?00-000?0?000??0?0?000?0?0?0??00?0?-??00?000000????????0110??
00?1?0?1101000??
Citipati_osmolskae
?011001001001-?221000101111?01011?00010210001100000110001000110121120-010
0010111-1---------1011101100101111001??201-?002100111?0111200110100100?110000
001000211001??01012010??10101111?2101000000000?00000000000000000001102000
0000-0-001000-000002011---0--?[01]000-01000121000100000001?102?1??0000--00000
10001010--0000020011000[01]0-00-0010000101020??????0????011000?0-0010000-000
000000000?00100?0??0?0??-0000000-???00000?2000000000000000000002-000?00000
00000100000110??00[01]10?0110110000
Rinchenia_mongoliensis
?01?0????0??????????0111?1???1?11?00010??00???000?0??????00??1?12112?-01000?
?111-1---------???????1???????????????0?????210??????????2??1???00?0???10??0001000
?1?????1????????????????1?????????0????0??0?0???????0??00??001??2?00??????-??-?
??????????00---?-???00???110??????2??0110?????????10-0?10--0???0?????????????0??
??
?????????00???0?00??002?0????
???????
Oviraptor_philoceratops
?01?0??????01?1????0??1??111?1011???0???1?0?11????0?1?0???0?11?121120-01?00?

01?1-1---------??????????????????????0?????????????????12??1?????00???1?0??00?00??
1??0????????????????????????????0????0????0??0?????00?00000???2?00?0????-??-??
?-10000?0?0---?-??0??01???0????00???0?00?????????10-0?00--0??00?00?1?1????00???
00?100????0????????01010?0-00-0?0????0?100??????1100???0????????00?????????00
0?0--00000???????????????????????????????????????0??000?????10????1?0???02?0??1
101100??
Conchoraptor_gracilis
?0110??????????1???00111?1???1?11?000??21000110?000?????1?0????121120-010?0?
0111-1---------?01010110010??1?012?110??10????01?1?????20011000010????????0010
0021000101010020 10??101001?1?11010000??00010000010?000??000?001102?0?0000-
0-0010000000002001---?-??0?0??0100012??????00000000?0????????10--?0100100????
????000??0?010??
??????????????????00-00??000002000000000000?0000?002-01?????????0?010?000??0
??002?00??????????
Incisivosaurus_gauthieri
?01?00?01?001??1??10001110100100 1?010?12000011000?11010110101111210100001
001?1?000210--00?0???
???00??00??01?0??0?0??
???????000000001000102-00010-?100??????00000000001?00001??0000--?0000101?11
00--0000???
??01010000???0??
000?0?0?????????
Microvenator_celer
???21?20?0????????
?????-???????011?0?11000111100???0?1-1002????????????00?000?11000??????00100
02???0000?????????????0110??0101100?0000010???????0??0???00??11?20?0??00??-0
?1??0???????????---?-???????0???0?2100000??
??0?0001????????????????????20000-000-01?????00?0-?010?00-000000000000010000
?????00????????0???0??000002000000000000?????????????0??0???????????????1?????
???0??????????
Chirostenotes_pergracilis
?????1??01?01101??0???1?110-0????????????????????????01010??????21120-00000?0
1???1---------???????1101?11????1?12???0?????????????????101?1???????????00?10002
1?0010101212010 1110?011???01??100???00?100000200000??000?0?1??20?0?000-0-00
100???????????---?--???00-0?????[12]????10????????1001????00????????????01?10--?
0????0011??00?????????????????20000-00000???????????????????????????????????????
???????????00000-00??00000?00?0????????000000?0???0000?????001010-00???0????

????1????????0
Caudipteryx_zoui
00110??????????????0?111??10?0001?10???21000???0?00?????????????21120-0?????
???0?1--0-----00????0??00????1???0???01-????20?????0???0??0???01?0???1000000?00
0?1??????0101201???1????101?11?????0????01000??1?00?0?000000?1102000??00?0-
001000000000?00?---0-??00?0-0000?1[01]1000000000???????????0??????0000????10
00--?0000?00?0000?0-0--???????????20-?0-0?000?10??00???????0?0???00?00????????
????1?-0000??-0000000-000000000??0?0?00????0000000??0-?2000000??0????????011
01100??01?110???0??
Dromaeosaurus_albertensis
?0??00100000000010??0???0?01??01110????1111????10001?1001100??00000010011
111000?0000101001???
??????1??????????????????????????????????????11?1?0????00??????00?0?00??1?
-???????1?00??011000100?0???0?????????????0???0000?000000??0000--000000000000
0--00???
???????00??????????????????????????????????????0????0?????????0101110000??0???
00?0?????????0?
Deinonychus_antirrhopus
?0110????1???????1??0000?01110001110001111111100?1????00001110100000?010011
1?1000?0100101000?110001100?1111011????00110111121??????1?110111001001110
000001002211101010102 20?01111201201?11110000000000100011010010000000000110
0000?00010-001000101000001111011 0000???000001210000000000?0?????000????0???
????????00000--000000000000?[01]0-0?????????????20000-000-000011000?0-0010000-
0000000000000101?0???00000--0100000-000?000000000000000000000000000-000000
00000????????0?1????00111?????????0
Velociraptor_mongoliensis
?011001001000012011200001011100011100012111?10?01000100001110100000000100
1111100010100101000011000110011111011111000110111121110010111111111001001
110000001002211101110102201011112112011111?00000000011101101001000000000
1100000000011-00101010100000111001100000000000001110000000000000?0000000-00
00--00000000?0000--0000?00000000[01]0-00-?000?00000020000-000-000011000?0-001
000???00000000000010100???00000--0100000-000000000000000000000000000000000-0
100000000010[12]1100001101100011100000?1000
Balaur_bondoc
??
???????????????????????1?1??1????0???10?????????0????111[01]11101001?11?00001
???0???0111?102?01?0?1?2122--???????????????2110001001000??00??1???????????0?

00??0???????????????????????1?????2?00??
????00000200?[01]???0-???????????20??????0-??0011000?0-001000???000?00?????01
0?00???21??0-00000020?100??0000??????00???0?000000000-00??0?00000???????010
????????1????????00
Atrociraptor_marshalli
???????????????????[02]00?0??1010??????????????????????????????????00??010????
????0?0000101001??
??0????0????????????
????11?00??0??111???????????1???????????0???????????????????????????????0????0?
???
???
?????????
Utahraptor
???????????????????[02]00?0??????????0?001?????????????????????????????????????
???0????0101??1????0?1100?1??1????????0??011??????????????101?1?????????????0??
???????0?01?????????????10?????0111?00??0?001000100???10????????11??0???????0
?????0?????????????1???0????????0???10???0???0????????????????????????????????????
???????00000????????????????????20000-000000????????????????????????????????????
????????????????00???????0??0000000000000?0000????-0?00???????????????????????
???????????????
Adasaurus_mongoliensis
?0110?????0????2????????????????0?????0?1??11?1?0?10001??001??0?????0????0?1???
0??????????????01100?11?0111?10?111[01]100??011?1?1??????1?111111????????????
???1022111010101?2201?1?11211001?111000000?000?11010010010??00??00110?0?0?
0001?-00100??1??????11????????00?00????1???????????0???????????00??00--?000?0???
????????000?0010000[01]0-???????????????200?0???00??00110??????????????????????
?????????????????????????00-000000000000000000000000000?002--10?00???00???110
000??0??0001?1?????????0
Achillobator_giganticus
??????????????????????????01?1??
??0000101??????0?01100?11110???????0??011?11??????????101?????????????00??10
220??11010002101?011010?001?21110?0????00?000?101????0???????1???0????001?-
00100011100??0??100?????????0??
???????0000?????????????????????20000-0?0-0?0??10???????????????????????????????
??????????????????00-??0?00000??000???????0?0000????????0????????????????0???????
???0??????????
Tsaagan_mangas

?0110010010000120112000010101001?10001211111000100011000010 0??000000100?
1111000101001010000 1100??1??????0?????????????????1????????11?111???????????
???00??00??00?0000??
?????????1110000011-00?00000000?00001????0000000????0000000-0000--000000000
0000--?000??????????????????????????20000-??0-0?0??10??????????????????????????
???0????????0111000???0
??00011?0?????????
Saurornitholestes_langsto
??111??????????????11????0?????????????
????100101?00?11000110011111011011100?1011?1?1????????????111??????????0001
11221?1?1?1?1?0?????????????????11??0????00??001101??00???00?0?1??00?0000010
-001000????????110011???????0????1210???????0?????????????????????00000?????
?????0????????????????????????????????0???00??1??????????????????????????????
????????????????0????????????????????????????????????0???00??????????0??????0
0111????????00
Bambiraptor_feinbergorum
?0110??001000012010[02]00?010111000?1100012111?10001?0?1?00011?0?0?000?010
0?1111000?010010100??1110?1100?1??100?0?1100[01]1011??2????0101011011110?0
01?1?00000111?211?0101?102201?111021100???1110000000001000 1101?0000000000
01100000000011?0010?010100??11?110??0000?0000000121000?00000?0??000000??0
000?-000000001?000--000000000100??0-?0-0?0?0?00000200000000 00?001?000?0-0?10
00??000000000?00010?????00000--0000000?000?000000000000000000000000?00-01
0?100000000111000011 0??00011101100-1000
Tianyuraptor_ostromi
??11??????????????????0????111??????????????0??1????????????????????????????????
?0?0?0?1010??0???0?????????0?0?0?1??01?0111???????1??00?011110?001?10100001
112?1??0?1101223?11111021?0???1?110??????????00?11????000??0???1??00?0??01?
?-1011?0???0??????1?0????????????0????1?000?0???????????????????????????????????
?????0???????????0-00-00????00000?0?00-000?0?0???????????????????????????????????
?????????????????00-1?0????10???????????????0?0????????10?101??????????????1?????
??????0000-10??
Sinornithosaurus_millenii
0011??????0???????????00???1110????1000111100?1???0?01??0????????00?00100??1?
???010100??100????????1??1???????0???00?1??1???????01?1?11011110?0????0?0000
?00?201?01?111023?1?111021?2?1???1?????????110001?1100?0?00010001??0000???0
1101111001000000111100?00?00?????00???10000?000??????0??000-???0????00000??
1000--00?00?0?????0?0-?0-??????00?0?[12]0000-??0-000000?00?0-?010?0??00????????

????????????0000--0000?0000??0?0?00????000??0?0000?00?0?-?0011?11000???????
01??10000111?1100?1000
Microraptor_zhaoianus
0??????????????????100???0??010?0?1???
?0?01000000????001?1??01?1100?01???0110?1212111?01?101111111010??11010000
11012111??0111023?1?1110211201?11111??00?0001110111110?100??000?11?000????
1010111100?0?0???1?110???0?00???0?00???10??0?000????????????????????????????
0?0???????00001000?[01]0-00-0000?001000200???0?000?00???10?0-??10100-000??1?0
?000?????????000???????0?00?0000000000???000?00??000000000-?1111111??00?01
10??011011???1?1?1100?1000
Graciliraptor_lujiatunensis
???
??020?--1??????????????????????????0??11?12????????????????01011?10100001????
?????????????????????????????????0??0?010?[01]11?1??0??0??00?????????????0????
??????????????-00????????????????100???
???0??????10?0???000??0????????????10?
0000?-00?000??10??????????????????????
??????????
Hesperonychus_elizabethae
???
???21
??010?????????????211211?????????????????????1????????????1??????????0??1?????
???
???
???0?????????????????
????
Pyroraptor_olympius
???
????????????????????????????0????????1????????????????????????001??????????????
???01??1?????????0?????????????0??????0?
???
???010??0????????
??0??????????????????????????????
????
Rahonavis_ostromi
?????????????????0???????????????011111???1?01?011112?12???????????0??11??011
???????0?01111?10111110?3?1012101?121??2111001000?011101101?0100????0??1?

????0??10001011111??
?????????????????0011000[01]0-??????????????2????????????0100????????????????
???????010100??????????????000000?000000000100000000?00000000-000??01???0
????????0?1?11?????1????????00
Buitreraptor_gonzalozorum
?0110???????????????00001011?00??????????100?10??0011?0?????????00??001?????
?????0210--100?0010111100111?110???100011012[012]121???????01101111010?1?10
??0??????11?101??11[02]23??01?1?1?120???11?0??0??1?011?0?1110000?????0??1???
?????1101000110000000???0?--???0000??00?00?10100?00?0????????????0-??????0?00
0101???0?????00000000000[01]0-?????????01000100?0-0?00??0?10000?0-001100??00
000100000001?100???????????????000????00???0?00??????????0?00?000-0000?00???
???????????1????0010??1100?0000
Neuquenraptor_unenlagia
??
??????????????????????11111111111???1????1????????????0??1001????????????011
11?1011111022010111?111202011?10000??0?010??111100000????0??11????0??11011
0?110???????????????????????0?????210???
????0?00000?0?????????????????1????????????0100?0?0-0011000-00000???????????
0??????????????00000?000000000000000000?00000000-00???????????????????????
????????????????
Austroraptor
?0100?????????????????????1?0????????12?0001?????????????????????0?011??????
?????210??10?????0011?001????11??????????????????????????????00?????????0???
????????????????????????????????1??????0?0?0?0??[12]1??????????0????0??????????
??????00?00??01?-00??????????????1[12]10??0?0???????????????????????????????????
?????0??00?00??????????????????????????????????????0?0-001100???00??0000000???
??
???????????????
Shanag_ashile
???????????????????1??1010110???????????????????????????????????00??011???????
???01001011???
???0????0???????????????
?10100001??-00??-?????????????????00??0??
??
??
??????
Mahakala_omnogovae

???????????????101??????????????????????1-00?????????0?0011??????????????????
???02??--1???00??0?11?011?111??1?10001101211O???????????????00??1110000O?10
0?211101-2?????????????????0111100????0?011001101?00000??00??11?0?????0000?
????00????????0?????????????0????1[01]1?0??????0?????????????????0??1?000????
??????0000010000[01]0-??????????????????????????00??[01]0???0?0000???0???????
??????100????0??0--?000000??0??001??0???000000000?00000000-000??00?000????1?
??0??????011?0????????00
Mononykus_olecranus
??????00???????112??????????????????????????????????????100???????????????????
????2??--0????????1?1?11101101?????10?2??????1???1000?000200030110-112201100
??1????1??000-0-1?????3??0--?2101001110112111000300000001??00???1??0?0??00?0-
0???00???????????????????????????10011??????????????????????????1????????????
????0?0010???????220-?-00?????2?-?0-0?0?0?001?0110-?0002000-00[01]-0000-000--00
00???300-0---????0??00????1??2000100000000?-0000?00-00?0??0?-000???????01-0???
??0?0?????????1
Shuvuuia_deserti
?0110100000000?112011000?0?0?0-111-1100210000101010010010???1?100001010000
02100?10211--000??0111111011101?0??1?201012012110100?1000?00020003011?-112
2011000?1??011?2000-0-10021032-0--?210100111011211[01]000300000011000001100
000010000-00??000000000001-0100-00000-0000?10010000000??101?00??1??00110-01
?1000??0[12]0---?0100?0010000-0-0220-0-?0?????20-10-000-0100000111??0001000-00[
01]?0000?000--???????300-0--000??00-0?0?00100200010000000000-0000000-00000000?
000001000001?01?1010001????????1
Patagonykus_puertai
??
?????????????????1?011?111???[01]12[01]10?2????????????????00200??0110???????
1????1????1??0??-??10????21101-?1100?000100001110??0????0?1??0?0?1???00?????
??-0010?0??????????????????????????????0???
??????0?0010????????????????????20?00-00-?01??????0?0-000???0-00?????????--???
????0????????????0???0????101200000000000??00?????????00??0--????????????-?????
???0??????????
Albinykus
??
???1
??????????????????????????????1101???11100300000????????????????0000??????
??
??

?????????0??????0????????010000100-0000000-?0??0????00????????0??????????????
?????11
Alvarezsaurus_calvoi
???
???????????????????100????????0?2?10?2?12?????????????0?0?00?????????0?????000
11??0??2????????????????????1?00???0??0??10000000???0????????1???0????00??????
?0???????????????????????0?????[12]???
?????0?12???00-??????????????2?????????0?0?0??????????????????????????????????
??????????????0??0?0?001??2????0000000???????????????0?????????????????????????
???????????0
Proceratosaurus_bradleyi
??1?0??????????????00010101110000?00??????????0???000???????????000?0000?00?
?0000000101011??
???00??0????0?0???????????
???00000010?1-00?00?00??-?0?2??????00?000???0???????00???????????0?????0?????
00??
???0??????
?????????
Ornitholestes_hermanni
?0100???0?0?00?1???0?010?01110001?100001?0??100000001?01011???000001000?
0?000010?00101001?????011?0101110000??000?0010??1???????????????01?00??????
????00001??1?0?0[01]00100000001??0?1???????0????????00?00???00000?0010?00?0
?0000?0-0000?0000000??01-0??0000000-0002?111000000000?0???000000-0?00--?0000
0000000---0000000010000[01]0-????????????????????????????????0?0-0010000-00000
0000000??0??0????1000--0?00?00-000?00000?0000?????????0000?000-000??00?0????
?0?000??0???0001?0?????????0
Coelurus_fragilis
???
????????????0??010010000111000?????0?000?????????????0?0??0?10001??0??0?????
?????????????????????01100?000000?0?0?00110?0?0????0?00??00???0??????????0?0
????0???????????????????????????11100???
?????001000???????????????????2????????????????00?0-0010000-00000000000001000
0???0????0-??0000?????????002000000000000??000???????????0000???????????0???
???0????????????0
Archaeopteryx_lithographi
101?0000??000??112010010??1110?011000012100?10?0000?0??100111?0?000001000
002?00000200--00100?1?1???00?0??1?0?0???0021012211000?-??1011111111000?1100

000000112111010?11003010121021?212?111100000?000100000030?00000000011000
00??00?000110010000001001-01000000??-?0000?020000?0000000??00110??00?0??00
0?00??000???00?000000000[01]000?0????????00000?0000?000000000000000?0011000
?000??000?0000100000??00000?000000000000?0?00000000?00?0?[01]1000000?00???
00000100001010000001011101?0101100?1000
Avimimus_portentosus
?01?0???10011?00??0??1?1???????1?-?1?????00?11-?0?00100110??????2?1???00?0??
0111-?---------?011010110101?1?00??100??????????????????????0100??????????00?
211?01??0101201010?010?0?1000100?0???00011110020???00??0?0?01?02?000000?0-
001000????????0?????????00??01??0?01001????????????????????0??0???00?00?????
?????02001000?????????????????????????????0?????0?0-0010000?000000000??????
?????2???????????[02]0-000?0000020?010000000000000??00-01??0??????0?????????
???????????????????
Confuciusornis_sanctus
10110??????????????1?000?00??0001???0??2??0??0??010???01???????000010000?10?
0001-1?--??????0??????????1011?0?2???0?2????3?-111?11010?-13111000?1111100000
01121?1?1-?11000?1?111022?21-12??10??11??120211010030?0000000001102000??00
?0101??11-000011001---?-?0??????000?122001[01]00110??????????10??00??011200??
01[12]011111000000110000100100012000 0?00?0000?0??0??000000000?000211??0010
000000010102101011[01]?10?000000101000000000101010010001100001 1000?00?000
0[01]000???1000001101110?00??1100?10?0
Struthiomimus_altus
?01010?110??0??1010210001011101-11000000000000000001?0??0001?01??0001000001
020001-1---------001?101100001010001010000000001001001?????011200020000000-101
00100000001100001010010000000001000001100010001000002 0--000011111110121110
000?0-0010000000000001---?-?00????0000??2110????0????????????0-0?1????000?????
0?00--?00?0000?0000[01]0-0-????????????20??0?0?000?0001101?0-0010000--00000000
000??0???0??00000?-000000000000000000200?00000000000000--00-020000000-1002??
0??00????0?00?00?????????
Gallimimus_bullatus
?01010?1101101010102100010 11?01-1100000000000000000100000001?0100000000000
1020001-1---------001110110000101000 1?100000000100100??????0112000200000?0-10
10010000000110000101001000000100100000110001000100000 20--00000111111012111
0000?0-0010000000000001---?-?00100-000000211000?0000[01]0010000000-0000--?000
000010200--1000000010000[01]0-0-????????????20-00-000000011001?0-0010000-000
0000?0?000?0???1??00000--0000020-0?0?0000020000000000000 1000-000-01000000?-
1002??0000?-0??00000?0????????0

Garudimimus_brevipes
?010?????01101?????2?00010101000??000?00?00?0?000??????0001??1?0??0??0??0?0
2?0?1-1---------??????????????1???1??0???0?
0???1???0?0????????0??0?1???????0???????000000000?0??01??1?1??2?1?0?00?0-0??
0001000000000---?-??0000-0000010???00?0000?001?000000-0000--0000010110000--00
00000010000???0??
?????????????00?0000000002000000010000000000003-010?0????0000???000???0??00
1000????????00
Pelecanimimus_polydon
?01???????1????????2?00????100?????????0??0??????????????????????00?0000?????
??000211--0001???????????????????????????????????0?00????2?????00??00-1010???
???001110????0?????0???-?0?
???000?0??0?1-0???-???????00?0?0?10???0?0???????????0-0?????????????????????0?
??????0???????--??????-----?0-???0?0???????????????????????????????????????1??000
0??-00000??????????????????????????????????????0??000???????????0?????0??0????
???????
Harpymimus_okladnikovi
?0??????????????????2?0??0??00?00?????
????200--1-????????????????????0?????????????????????????????2?000000000010?10?
?0???????010?????00????0?????????????????0000000-??0?001011????1?0???00?0-0??
?000??0000??1-0????????--000??11?10000000?????????????0?????????????0000--00?
00000?0000[01]0-?-???????????????????????????00??01?0-0010000-000000000000---1?0
10-000000-0000000?0?0000?00?????00000000?00000?00-?00??0000-1????????00-????
?000???????????
Troodon_formosus
???1?1112-1101000001???0?011?0??????20220000210?00?1?01100????0?10??001????
??????0111010100???11111001011111111?1000?1020??11?????????????1?010????0?00
0010??????????0?0?2?10?11000?0??01111000???00010000021??01????000??1?0??010
???1-?0??0?0?00000?0?0???????00???????????????????????001??0-000?????0??1?1?
????????00??
???0?????00000101001??0
???1110?1?????????
Saurornithoides_mongolien
?01??1?1??1101???0?110001?1000????????2??????????????????1?010?100?0010??1?
???0001110101?????????1???0??1?????100??1????????????????????????????????????
???????????01012010?010??10?1?11110???????????0???10?01??????0??1?0?????????
??010??00000??0?1-01?0-????0??000??????0??0000?????0?1000-0??0--?????????0000-

-?0????00100?0??
???????????????00-???????0?0????00000000000000?20-0200000000000101001?10???
1110?1?????????
Saurornithoides_junior
?01101?12-110100?001?000??100000????2022000?21??0????11100??????100?001???1
????000111010100?????????????????1?1000?1020?1?????????????????????????????
?????????????1?????????????????????????????011????2???????????00??00???11?????
???0??000000000100000-???1???00???????00?000??????001??0-00?????????????0000-
-0?00????????0[01]0-??
?????????????????????????????????00010000??000??2????0??0???00000101001?11?
??1110?1?????????
Xixiasaurus
???????????????????01000101110??????2?22??????????????????????0?00??001??????
??000201--01?0??00?1???
???0???00????????????????????
?0?000000??-01?0000?????00???????000000???00????????????????????????0???????
??0
??????0??0?0?????????????????????????
???????
Byronosaurus_jaffei
?????101???101?1100110001011?0??????20220?????????????1?100??????0000001??11
????000211--01?0?0???????010111????????0??02???????????????????????????????????
?????????????????????????????1????0?0??0???????21??????????0????0????1??????
?????00000000?1-00????0??00?000??0???000000?????????????0??????????????0?0---?
0????0010???
???????????????????????????000?????????????????????????????000100011??0???????
?1?????????
Sinornithoides_youngi
?0??01??????????????1?000??1??0????00???2???????1?0??????????????00??0010?????
??0001110?01??????11??001???????????0011?10112101????1?00?1?101?0???1000000
????21??01??01??30???11?0??001??1110?????0???1?000110001?0??0?0?1??00?0???0?
?-0010?0??00000???-0??0-?0????00???0?1000000?0????????????????????00?????1?0
0--000??????????[01]0-0--------0??0?20-?0-00000?0??1000???0?1000???00?0?????????0
??010-0000??-000000??00??0??000????0000000000000020-0200000000????????101
??????1?010100??1??
Sinovenator_changii
?0???0002-000011110010101?1110?011?02??2??0011???011?10100????0?000?001????

??0000011110?1????11?10100011110?001000110221?1?????????110111???????1000?
0???0?211?010111[02]0301?1110211201?11110000??10011000111??110?000000???00
001100010001100000000??1??011??0000?000000??0??00000000000??000000-00?0--000
0010100[12]00--00000?0010000?????????????????20000--00-0100?10???????????????
?????????????????????????????00-1000000100000000000000?0?00?020-0100?000000
000100010?10??1011000???????00
Mei_long
?0????????0????????11010?????0?11?002012100001?10?110??100??????00010010??1
?0000?021?--01?0011011100011?1110?01???0110221111?11????111?11100?0?1?1000
0001?012111?1??1100301?1110211???0111100?0101001000??110011?0?0000???00000
??000100?1??0?0?000000?001?0?0000??000001010000?000??????????00?0??0--00??0
101????????0?0000010000[01]0-0-???????0100020-00-00000?0011000?0-0000000-0000
000?000?01?1?0???00000--??00000?00000010000??00000000000?000021-01000000??0
???100??1010??101100?1100?10?0
EK_troodontid_igm_100_44
?????0012???1??0????????????????1??11???
???????1??????0???10?00000???
??01?1?001???00??????0?0??1?????????
0??1?1????????????
??
???0???
??????
Jinfengopteryx_elegans
?0??????????????????1?01???2000????????1??00?0????01????100??????00?00??0??1??
0?000?1???0???1???????????????????????02????1?200?1----?1101110??????1000000???
??1???????????????????2??0???????????0??????????????????0?0??00????00????????????
??00?000001???0??0-0???????0????100?0?000?????????????????????????????0??????0
?000???0?0??0-0???????????????00?0???0?0?00???0????????????????????????????????
???00?0000???????????0??11?01??
???????????
IGM_100_1126
?0????002-0000?11[12]01?010??2000?011?020121000110?0?10010100??0??000000000
?11?000000211--00?0????????????????????0????01102????????????????????????0??1000
0001???211?010?010020100010121012??111000????0??2?001210001?0000011?00?0?
020000-00100?0000000111-01?0-00?0000003??????000000??????00??00-00?0-?0??000
0000?00--?0?0??????????0??
????0?00???0000??-0100000-00??0???0?00?00?????00?1?000?20-02????000000101001

11??0??101?001???????0?
IGM_100_1323
?01???????0???11???1?000??20000?1??020121?00?1??0???0??????00?0?000?0????1??
00000021?--0000??????????????????????011??????????????????????????????????????0
0??1??????010?20100[12]102?1011???????00???0????0??2???01??00??0????0?0???00?
0-00100?00000000?1-01?0-00?000?003??????0000000?????00?000-00?0-????000000?0
00--0?00??
??????????????????00-????00?00?0?00?????????1000??2????????????????????????????
?01?????????????
Anchiornis_huxleyi
001100?????????????11010??11100?????0?2?000??1????1?1??????????-000??01??1??
???00020?--01??0?101??-00?0??010?01???01101210??011----011111110000??10000000
10?21?10011010?201?1?112112????1?1?000??1?01?000121100010?1000?1??00?0????
???0?11?00?000001??-0??00??????000???020000?000???????????0-?????0????0??????
0??????00000?0000[01]0-0?-------0000020000-0?00??0?10000?????10?0???0??????????
??????10?01000--0?00?00--00?00000????????????00000?0?00??0000000000????????00
??1110?1?1?1100010??
Xiaotingia
?01??????????????????1?1???111000????0?2???????????01?1?????????????001001??1???
00??020???01??0??0???1?0?????10?01?????1??????10?1????01?01111?10???10000001
011?1110?1101122?????102?12????????????10????????1?010001010000?1??0?10??00?
??0010?000000??1???00??0?????00?0???21000??000??????????????????????0000????
?????0??000?10000???0????????010002?????????????01?000?0?00???0???0?0?0????0?
??????????01000?1?00000???0?0000????????????????????0??0?02????10000????????0
0??1??????1?1100?10??
Segnosaurus_galbinensis
???1??10?0000?00
0???01000001?????????????????????1???0??????????????????????11???00????????010?20
021?0000?1100211?0?1020?0?????????00??0?000?0000?21?0??00????1??0?0???00?0-
00--0?????????????????????0??????????0?????????????0??????????????????????0?0????0
??????????????????????????????0??????0????00???????????????????????????????????
?????????????00-?000???10??????00??0??000000000-?2??0????00???????????????????
?0??????????
Erlikosaurus_andrewsi
?0110??02-0-1-0??1010011100-?0001?100000100001000?0??00000??111121001000000
20001-001000001-???0?????????????
??0?00?0??????00??01??00?0?00??????

?????-0-00001010010--??0000?000????0?0000000000011---10-0100--?000000000000--0
000??0-0010000-001000?000?????????
??0???0000-?0?????0000?0????000???0?000?
0???????????
Alxasaurus_elesitaiensis
??210?100???????
?????100?001??????????0?01010000?1?000??02?0?1??????????????00000?100-000012
0021?00?01010?211?021?2??????01????00????????00000210000??00??1??00?0??00-0-
00--00?????????????????????????0????0???????????????????????????????????0--0
0????0??00??[01]0-???????????????0-???????0??00??????????0?0???0???????????????
????0000??-000?????????????????????????????????????0000000?0????0???0???????
???0??????????
Tyrannosaurus_rex
?10000?0110000100210000010101000000011010010211000010010001?000000000100
11001000000000101011010000?00001010000001000?001000??1?????000000010000?
?0-?100010310001000000010110110000001?010010000?0001000002000000010000000
000000000-0-00101001000000011000?0111110111121020110000000000000000000-0000-
-0000010110000--0000000011000[01]0-0????????0000020010-0000010001101?0-00100
00-00000000000010??0????-000---00??00-000000000210000000000000000000-00000
000??02000??0001-0??00100000110?0000
Tarbosaurus_baatar
???????????????????0???????????????????1???????????????????????????????????????
???0??????????????
??2?????????????????????????????????0?
??????????????11111011112??2011000000000??00??????0000--?000??????????????00?
??-?
??000000-?02000??0001-0??00100000
???????0
Albertosaurus_sacrophagus
?1000??00?0000?002?0?00010??100?0000110100-021?0??0?00?00000000?0??????0?1?
??000000001010111?????0????001?10??0????000??0????????????000000010000????01
000103100010000000101101100000??0?00?0000??001000002000000010000000000000
?000?0?0010?0010000011000?00111101111210201100000000???00000000000--0000
0101??000--?0000000??00??0?0????????????0?20010?000010001101?0?0010000?0000
00000000010??0????-000???00??00?0000000002100000000000000000?00??0000000??
02000??000?-0??00100000110?00?0
Gorgosaurus_libratus

?10000?00?0000??0?10000010101000000000110100-021100?01001000000?0000000100?
1001000000001010110100000??000101000000100000000010000?1000100000000100000
00-0100010310001000000001011011000000100100000000?000100000200000001000000000
00000?000-0-00100000100000011000?0011110111121?2011000000000?000000000000-
-?00001???0000--?0000000??00??0?0???????????0020010?000001000110 1?0?0010000
?0000000000000010??0????-000???00??00?00000000021000000000000000000?00??0000
000-?02000??000?-0??00100000110?00?0
Daspletosaurus
?11000?00000001002?00000101010000?00110100102110000100100010000000000100?
1000000000001010110???000???001?10??0????000??0????????????000000010000????
01000103100010000000101101100000 0??0100?0000??0010000020000000100000000000
000000?0?0010?0010000000011000?0111110111121?201100000000???000000000000--?0
0001?1?0000--00000000??00??0?0???????????0020010?000001000110 1?0?0010000?00
0000000000010??0????-000???00??00?0000000002100000000000000000?00??0000000
??02000??0001-0??00100000110?00?0
Eotyrannus_lengi
?????????????????????000?0???0??????100????????????00???????????00??010???????
?0000001010?1???00000001?0?0???????????
??????????????????????????????????????0????0??????0????0?00????00?0?????0????
??????00??0??00??0?11?????10?????1?0000??????????????????0--0?000000???00--???
?000????????????????????????0000-0?????000?0?0?????1000???000000?????01??????
???00??0?????????????????-?????????
??????????
Dilong_paradoxus
?0100??0000000?100100010??1010000100110100001000000010?0000??????0000000-?0
0?000000000101011100 01?0?0100??1?0??????0??012[01]0??????????00000011000???
??0000?00001??10??0010100?0?0?010000000000?00??00010?00100?0000?00000?0?00
000?000?0-0010000000000011100?000111--?00001010?000000??????0000?0-000???00
0001?100000--?000??00?000????0??????????????20-00-0?0-000001000?0-0?1000???000
000000??0?0??0????1000--0000000-??0?00?0020000000000000000000????-??0000?0000?
0?10000???010000000?????????0
Shenzhousaurus_orientalis
???0?--??????????-?210001-1000??1???000-?00?000?00--??????????0-00000000?-0--0-1
-12-0--1----------??0????1?--0?--00000??[012]0???????????????????????????0010000001-?
110-0000100?000000100?-00001?0??????????????????0??1??1?0011?????00??-0010??
00000010??-???-??0??0??00???0???00?000????????????????????00?????0??????0?00
?0?100?0-0-0??0?

???-0?00000-00000?0002?000??????????????????????????00??????????????????00?00??
????????
Ornithomimus_edmonticus
?01010?110-101?1010210001010101100000000000000000010000000?????000000000
10-1001-1-------0-001-1011000010100010100000000001001001????011200020000000-10
200100000011000010100100000010010000011000100010000020--00?011111110121111
0000-0-0010001000000001---?-0?0100-00000001100?00000?????????0-0?10???000000
0?0??????000000010000[01]0-0--------??????0-?0-0?0?0?00?1001?0-00?000???0000000?
?00??????1??00000--0000020-000?000002000000000000?0??0????-0??0??000?1??????
?0???0??0000000????????0
Archaeornithomimus_asiati
???
?????????????00?101100001010001-100000000?0??????????01?200-20000-00100[12]0-
??000?110000101000?00?001001000001?0001000100000?0?00001?11??10??1?1??00?
00-001000???
??????0?001000?[01]0-?--------????20-00-00000?0?1??01?0-0010000-0000000000000?0
000???0?0000-?00??00-????00?0020000000000000?0000??00-0?00?000??????????????0?
?????0????????????
Anserimimus_planinychus
???
?????????????????????????0??????????????????0???????????1???0?2????000?10200100
000011000010100????000?001?????????????????00002?-?00????11??10??1?1??00?0?00
1000???
?????????????????????????????0?????????0??0??????????????????????????????????????0
10000-00000??????????????????????????10000??02-?0??0??00?1??????????????????????
?????????
Huaxiagnathus_orientalis
?01?0???????????????0001???1???0?????00??0?????????????????????????00?0010??????
??00010?1010?00??0???1????????0??????00???02100010--??000000000?00??00100000
00020?0??000000100?000011?0???0??????0??0?010000000?0?011??000????00?0??00?
0-0010?0000000???1-00??-?0?????000???100???0000??????????00???????????????????
?????00?00?1??0?[01]0-0--------0000020-?0-0?0-0?000100??????10?0???0?????????????
?????-010??--000??00-0???00000??????000?0??00??0??0????000000000????????000??
??00000?0?00??100
Sinosauropteryx_prima
001?0???????????????0001???1???0?????0000??????00??110????????????00??01???????
??0001001010?00??01??100????1?0?0????00?1100100010--???00000000?10??0010000

?00020??????0000100?000?11?0?1?00?0??000?0?0100000000?011??0000??000?0??0
0?0-000??00000000??1-00?00??????000????10000?000?????????????0?0???????0101?
???????0000?0??0?00[01]0-0--------??????0-?0-0?00???0??00???????0?0???00?00??????
??????1?-0000??-0000000-0?000??0?2?????00??0??00??00?0???000000000????????0
00?10???000?????????0
Compsognathus_longipes
?01?0???????????????0010??1??00?0?00000????011???0??000????0????00000100?1?0
0?000010010101000?01??00[01]0???1?0?0???000?010110001?--???0?000000?10???0??
?00?00?????????0000100?000?01?0??????????00?0001000000000001100?00????0?0??
?00?0-0010000000000001-00000000?00?000?1010000?0000??0?00??0?0-???0--?0000??
???1?0--0000000?10000[01]0-0--------00?0?20-?0-0?0-0?00?100????0?1000???0????0???
??????????-000???-0?0??00-???000?00??????00??0??000?00000-00000000000????????
0?????000000????????00
Juravenator_starki
?011????????????????00000??11000?????00021?0?00?0???????00???????0000010-??0?
???000100101??00??0????????????0???????00??100?0?01?--??00?000000000???0?0000
000000?01100????????????????????0?0???00??????1?0??000000?10?000?1??0??0??00-
0???????000000000?1-00?0-000?-?0000???11000?000????????????0-????????????????0??
????000?00???00?-0-0--------000002??00-??????000100000-0010000--000?000?000??-0?
0????100??-0000?????00?000??2??00??????000??0?00?0-00000000000????????00????
??0?0000000-10?0
Jeholornis_prima
10????0????????1???0???0?????001????????????????????10????????????0000-1?-?00?00
?1-1-------?-0????????0????1?0?11???02111021??01?0???011031110001??1100001011
?1??0??01?0???1???1?211?10???????01????010110100300000000??0??1000??00-010
????1???000?1??---0???0??0-??0??12200?10010????????????????????0????00000000--0
0?000000100000-0--001??-0000?00000?0?1000110001000-0010000-000?0000?0000100
0010110000-000000000000?00000?010000000001000001000?000000001100????????01
1?1110???1?1100?10?0
Jixiangornis_orientalis
101???????????????????0010?????001????0?0??00000????????0100??????000001????0?
0??1--0?-------00?0???1?00????1??20???02?-1021??11?1?00011031110001101000000011
112101010211000010101?211?10?2-11?001??1?01[12]1101003000?00?000?0??1000??0
0-0100??110?001?1-??---???00?00?0000?12200?00010??????????????????????????0?????0
00--00?0000?11?0?10-11?0?????0000000000-001001110010?0-0000000-000???0??0??
??0??010?00100--0000?001000000100??0000??00000100000?00-0000-001100????????
00??11????01?1100?10?0

Yanornis_martini
?01?????????????????1?010?????0?11???0??2??0???????0?0???????????0000011-??0??
??000?1???00??0????1???0????????4?1???????03??????110?011031110?011?1130010?
???????????????????????22?010?2-?10??1??0?0121[23]0000300000??0001???00?0????
???0???21?0?0?????????????????????????20010001 1???????????10????????1?????????
????????0?0011?00???0210?[12]??10100001010?1?11?111100111??0101?0101?10000?
0?????021[01]101311[01]1100[01]100?0?1?0??0?00??????0??00?1110[01]?1?00??1?0??
01100????????01??1????????1100?11??
Apsaravis_ukhaana
????????????????????????0???0002?1-0??0????
????--??--??1??11??10200??1???5?100021??23??????1?0101103110000111113003--000
?1???1-200000-1?021?2220--12-010?????1??12130100?00000??20??-1?20?0???--0-00--
21?????????????????????????????200?10??????????????????????0?1[01]??????1?0????
????20010?[01]001??21??1????????00000?0?1000111011111?01011010[12]011111000
0101021110?[23]120100?11??20?1[01]0010111?110?1012112111111?0000?1-0?-01101
????????011??????0?1????????00
Yixianornis
101?????2-021??1?????0?01????0?1?1?0??????????????1????0100??????000?010???0?0
0?00120?--10?01???1???0000??1?0?40??0?-?0023???11?1100011031110?00101130000
100021?101-21100301000102222--0211?0??1??????2130100100000??000?0??1000?20?
-0?000001??????????-0??0??0????0??0?1220010001????????01??1?????????11[01]?????
1?0?????10?20011000?11021??[12]??10100001010?1111011100111100?0110???01?0
00???0?01021[01]10?31201100[01]100?00100?00?002?101??????11110[01]?1?00??1-0-
001100??????00011?11?01????0100?10??
Sapeornis
?011?????????????????0?010??11000????00??21?0?00??????0??????0????0000010-?10?
00000020?--?-?00????1???000????0?211??0210003??100?----2110111101001110000001
01121??00-211000-101[01]00211210????1??01??1001210?1003000000000????2000??
0??0100--0100?000???1-??00-?0??-00000??22000000000??????????????????????????0
?00--?000000001000 0100???????-0000020000-00000?000001000-0?01010-0010000?0??
?010?1[01]10?11100--0[01]00?0000?0000000?000??00?00??000001?00-?0-00001000???
?????001?1?1011?1?110001000
Neuquenornis_volans
???????12-???????20?????????????????????-??00???????00????????????????????????
??????????????????????00??????????????????????1?11011?1031110?011111000??????
?????????????????????????????010????????????[01]010030??0????0??????0?00???????
????1????????????????????0????????0100???

?????0110???????21?????10110??0100?001?001?0??[12]??1???01?0???0?????[01]????
?????2???20?1??01????????????????10?1????????1?0???1011???????0?000???1000001
10??????????????????
Patagopteryx_deferrariisi
?0??????????????????????????????????????-0010?????????????????????????????????
?????????????????????2??0?1????????????21
1101?????????????????????2-010001??100121301001?000???????1????????00-0?????1
???????????????????????0?????0???????????????00?1??????00??111110000????????
010000000????????0????????000????0101?1110[01]00???[01]?10?0???01000001[01]00
01?[12]00???30???????00?2000000?010120????0?000[12]11001100000?[01]?0-?0?100?
1?100??01?0???0?001????????00
Cathayornis
?0?????????????????1?010?????0?1??????????0????????????????????00????????????
?00001?--????0????????[02]????????3?????2????3???0??11?02?1031110?011?11300?0
?01111?0?00?11000-101?102122--????????1???0??[12]1[01]1?0?3000?0???00????0??1?
?0?-0100-??100?????????0??0??????????????2200[01]0?011????????????????????????
???0??????0????0111??0010?20??1??10110?101????010?01100010010110[01]?010201
11110?[01]?1?????210120?11?01?0101011?0?000001???1?0?0?1[01]1?0????????????0?
?01110????????01????????01??????????
Concornis
??
???????????????????????00????????????????????0??11?02?1031110?0?1??1?0030-???
????????110?101??11?222?--0???1???1???0?1[02]11??0?3000?0???0?????????????????0
01??1?????????????????????????????2200???
?????0111????10?20??1?0101100101?0?0010001?0??[01]001?110??0102?1???10???1[0
1]1?[12]??????????????[01]?????????????00?????0?100??1?00??1?[01]1??0??????110??
???????????????????1100110??
Gobipteryx
?0??????????????????1-01010?0?0???????????????????????????????11?000?0?????????
?1-1-------???2???????????????????????????
??????????????????????????????????????11???????????????????????????????????1???
?0?????????????????????????????1100100?001?10?10?????00?????0???0??0???1??????
???????10??????????11??10000?0?100011000[12]001?1?0110???0?????????????1?121?
?[012]0?????1?0??1????????000?0???10?001111000?1?11?0????????????????????????
????????????????
Vorona
??

??
????????????????????????12-0100111010011121110???00????????????????????0?????2
??
??
?????????????????????????1010111100001?000000???????????????????????????????
?????00
Songlingornis
????????????????????0?0???????????????????????????????????????00?????????????
000?0????0??????????????????????????????????????11??[01]???3???????????????????
??0????0?????0?????00?????????????
????????????????0??????????????????0?01???????????????????????????????????0???
????????????21??[12]?000100?010???1?11011?????????????????????????????????????
??0-?0??????????????1??1???????
??????????
Pengornis_houi
?0110??????????????10010??0-00??????0?12??0???????0??????0??????000?001??????
??0?0?00??00???01?????-20???0???2????--????3????1?????2?1031111?0???????03--00
1?1???????????????????2??2???????????1??????211???0?000??0??000???0??0??00???0?
??11-0-000?????????-00-?-??00001220000?000?????????????????????????????0?????
0???0011??00100????????01[01]00?00??????0??1??012111[01]0?011010001[01][01]1??
???1??1???10[12][12]0??1??1?11??????0????00?0????0??0[01]?1?00??1?11??0-0--0?10
0????????01?????00?01?1001011??
Hesperornis
?0?????????????????00010100-00??????00021?001????00?0???00????1?000?001???1?
0001-021?--00?-10101110-2100??11?50100?210?23??11??10??00103111-----------3--020
2100-1--00000--?02102220--12-??0001??11?1213110010--0-00--01-1?0000?000??-00--2--
0-1111-???0??--???10-000??10-1-10?11112001?10?1010??1110011100??00?0???101012
1011000012?0?000100100?0???10?0???00001??0?????????0???0????0???????????????
???????????20?1100001112110211110021111122122002?0-?---00??????0001-0???0?00
0?0100?11??
Baptornis
??
?????????????????????2????????5?????????23?????????????????????????????????????
???????????????????????????????????????212???????????????????????????????????2??
??????????????????????????????[12]????????????????????11001??00??1??????????1
21011000012????012?0?????01?10?0???000????0?????????0???0??????????01?[01]1[0
1]???????????????20?1100001112110211110021111?21020?2??????????????????????

?????????????????
Ichthyornis
?????00????????1220????????????????????000111???1?10???????????000??1?--??100
0??0210??10???1-01111?2100?1???5?000?21??33???01?100?001031110?0111113003--
0102?1??10211000--??210222---12-?10011??11?1213110???00?0??20?????00????0??00
??--21??????????-0??????????????012200[12]0?11??????0?????10[01]1?111011101001
020???101?1200111?101[12]?211022010100001010?1111011110111100101101110100
00101[01]10112111123120110011?12001[01]0010111211021111002111[12]2?100002-
0--011-100?10???0110??1??00?00100?11??
Iaceornis_marshii
??
???1101?010311??????1113?03--????
??????????????????????????2??1??1???????21?????????????20???1??0??????????????
??
???????????21?02[23]11???1101010?11110111101?????????????????????????????????11
131401110111020010??1?1?12110212110021??????????????????????????????????????
????0100?11??
Limenavis_patagonica
??
??011?113?????????
???20?????????????????????
??
???10000[01]010101011?11[1
2]313011?011??
????????????
Crypturellus_undulatus
10----012-020002?2001010100-00-111-1200210000101111000010---1-10000-000-?10??0
01-1---------1110111122100?01107-110--12-23--121?110100103110110111113003--0012
10001-20120111002102222--12-01000100110121301-0---01000200001-20000200-0-0010
21---120--01------0?-???00000102002112111[01]0101001111101011001101111111111001
0101210101121121211112?1010002101101011111110011111011000120110000111010
1011111031401011111020?120110111211020211002111122210010100--011-10101000
001111110000??1100?10??
Lithornis
101????????????????01010100-00?111??2?02?00?0?????????????--?-10000?00????0??
101-1---------1????????2????????[67]?1????????????[12]??1100001031110011?113003-
-000210101-20100101??2102?????12-01000???11?12130100----?0??200?01?2010???0-0

-0-??21???12??????--?--00-???00000??200?11211100?010011111010110?11[01]1101?11
11?01?10121012?0111[12]121112211010 0?0101101111011110111110011000 1[12]1110
0001010101011111131301 10[01]11102[01]01201101112110201100021111222100 10[01]
?000011?1????????011?111??00???????????
Gallus_gallus
101--?102-021002?2000010100?00-111?120021-0001-101000001----1-1001000000000?1
101-1---------101[01]1111221?0-01007?120?-12-23--1[12]1-11012010311020011111 3003-
-001211111-2-100111002102222--12-0100011011??2130100100010 00200100-20100200
-0-0---21---121--01---0--00-???000001020011021112111111221101111111111110110001
1010111210101120111211112110100021011111111111100111110110001201100 00101
11010111111314010012110 21?1211111112110202110021111322200111-00-011000101
00000111111-000??0010110??
Crax_pauxi
1011011?2-021000??001010100-00-111?1200210000111010000 0100--1-1000000000000
?1101-1---------1000111122100-111?7?120--12?23??121-11002010311121011111 3003--0
002100?1-2?100111002102220--12-0100011011?1213010010001000 2001-1-20100200-0-
0---21---121--01---?--00-???000001020021021112111111221101111111111111011000110
1011121010112011121111211010002101111111111110011111011000120110000 10111
010111111314010112110 21?1111111112110202110021111322200110000001100 0?010
00001111110000?11100011??
Anas_platyrhynchus
101--?102-021002?2101001100-00-111?10002??00?1-10100010?0?--1-1020000100?01
1101-1---------101-1111221?0-011?7?11?--10-2310121-110111103111110111113003--001
2000-1-2?100111002102221--12-0100011011?121311?0--?010002101-1?20100-00-0-0-1
021-0-121---?---?--00-???000001120021021111111111221102111211111110110001111 0
1112101010201112111131201001010 10?1[01]1111111000111001100012011000010111
0111111123140101011102 1?111110111111010211002 1111[23]2210010200-0011-10?01
00000111111?000??0100?11??
Chauna_torquata
001??1012-0210002?101010100-00-111?100021-0001010100010100--1-100000000--001
1101-1---------1011101112100-111?7?11?--1012312111-10000103111110111113103--00
12101?1-2?100101?02102222--12-0100011011??213010010000000200101-20100000-0-
0-1021-0-121--01------00-???000001220011021 11[12]11111022110111121111111011000
111101112101210201112111140101001010111111011111102111001100012011000010
1110101111123140101111102 1?1111111112110202120021111222200100000000110001
0100010111111 0000?11101?11??
Hongshanornis_longicrest

101????????????????11010?????0-11???0????00??????1??????????????000-001-?00???
?1-1-10---0?-0??01??1?0????????[234]??????????3???1??11?01110311100011111130020
?0112???01-20????????2??2222--?2-?100?1??????21[23]0100000000?000?01?1??????
?-0?0????1?0?0???-??---?-?0???--0000?1?200???011???????????????????????????00??
????1?????0?????110????????01?0??11???0?10???11000111???110???2????0?????????
???1??2000??000100?0??0?0??000????????1???1100??[01]?00??2-0---1??????????????
?????????0100?11??
Liaoningornis_longidigitu
???
???3??1??0?????????????????
???????????????????????????100?1????0?21211003000?????0?????????????-????????
????????????????0??
?????????011?01??????????0???0????????????????????????????0?????01???2???????
??????????????????01?1?0??0011111110201000?00??????????????????????????????
???????
Epidexipteryx
-00???????????????????1??????00?????0??2?-0011???????????????????100?0?0????00
001020???10000????????????????0?[01]????1?1?122??10??0????0101?010?0???????00?
1???????????012?????011?02?0???????????0????011?0??0?10?000???00??1?1?0???00?
0-00--?0????????????1??2?????0????????1?0?????0????????????0????????????????????
??000?0????00??0-0???????????????0??0-0??-???0???0??????????????00?[01]0?????????
???????????????????-????????????????????????10?0?0?0????00-00???0??????????1??11
??1?????????????0
Haplocheirus
?00110??0?010?????01101110111100??10??20?00-??100?0???0101?001100001011011
0?1000?011?100??00??10??0-0111101??0??0001?0??20??0???????100?100??110?0??0
0010???1???00-100102000010?011000?10000000???0??0000000??00?11100011100?00
01?0-?-0010?00000001000????00000?0??000?10??00000000??????????0-???????????1
0??0??0--0000?000??00??-????????????????0?00-0?0-0?000??0??????12?????000?00???
????????????0??0?0-0000000??00?000?0???00?00??????000?0??????0000?000????????
?-?????00??01???????????
Fukuivenator
-0100-1001010?1---12-01-001-1-----------?101--0-1----01001----0-----0?0--------0-0200--0--1
1-11101010011?1001110-0010100100---------0001001-01-0----00011---------------------------0
0-00101-0-----01000000100000---00---000--0-0--01------0101000201--00--0----------012100-
--0----0----01------0---------------------00-00000000--0----------------00-000-0100--00------1100--
-0100011000000-000---000000000--0----------0-00002000000-0000000013-01000000-00-1

-100-11-00---1010----------0

5. References

Goloboff, P. A., Farris, J., & Nixon, K. C. TNT, a free program for phylogenetic analysis. *Cladistics* **24**, 774–786 (2008).

Shibata, M., & Goto, M. Report of the 3rd Dinosaur Excavation Project in Katusyama, Fukui, 2007. *Mem. Fukui Pref. Dino. Mus.* **7**, 109–116 (2008).
